# Effect of ketone monoester supplementation on elite operators’ mountaineering training

**DOI:** 10.3389/fphys.2024.1411421

**Published:** 2024-09-03

**Authors:** Toshiya Miyatsu, Jeremy McAdam, Kody Coleman, Ed Chappe, Steven C. Tuggle, Tyler McClure, Marcas M. Bamman

**Affiliations:** ^1^ Healthspan, Resilience and Performance Research, Institute for Human and Machine Cognition, Pensacola, FL, United States; ^2^ Department of Neurosurgery, University of North Carolina School of Medicine, Chapel Hill, NC, United States; ^3^ School of Health and Human Performance, Dublin City University, Dublin, Ireland

**Keywords:** high altitude, hypobaric hypoxia, cognitive performance, resilience, military, β-hydroxybutyrate

## Abstract

**Introduction:**

Special Operations Forces (SOF) often conduct operations in physiologically stressful environments such as severe heat, cold, or hypoxia, which can induce decreases in a variety of cognitive abilities. Given the promising empirical demonstration of the efficacy of exogenous ketone monoester (KME) supplementation in attenuating cognitive performance decrease during hypoxia at rest in a laboratory setting, we conducted a real-world, field experiment examining KME’s efficacy during high-altitude mountaineering, an austere environment in which US SOF have conducted increasing numbers of operations over the past two decades.

**Methods:**

Specifically, 34 students and cadre at the US Army 10th Special Forces Group Special Operations Advanced Mountaineering School (SOAMS) participated in a randomized, double-blind, placebo (PLA)–controlled crossover trial (KME vs. PLA) over 2 days of tactical mountain operations training. The participants ascended from 7,500 ft in altitude (basecamp) to 12,460 ft on 1 day and 13,627 ft the other day (in randomized order), while performing various training activities inducing high physical and cognitive loads over 8–12 h, and consumed six doses of KME or PLA 2–3 h apart throughout each training day.

**Results and Discussion:**

While KME increased blood ketone levels and decreased glucose levels, there were no clear indications that the elevated ketone level enhanced physical or cognitive performance. KME also produced a greater incidence of heartburn, nausea, and vomiting. In these elite operators, high-altitude mountaineering had a limited impact on cognitive performance, and KME supplementation did not demonstrate any benefit.

## 1 Introduction

Special Operations Forces (SOF) conduct extended, long-range patrols, which demand intense physical and cognitive exertion, often occurring in physiologically stressful environments such as severe heat, cold, or hypoxia (Shaw et al., 2021). In studies conducted with military personnel involved in field training exercises designed to simulate combat operations, decline in a variety of cognitive functions have been observed, including vigilance, attention, and memory (Lieberman et al., 2005; Lieberman et al., 2006). Therefore, there is an urgent need for countermeasures that enhance resiliency or augment operator performance during missions and maximize the efficiency of training by mitigating the deleterious effects imposed by these challenging environments. Mountainous terrain is one of these challenging environments that had a high demand for US military operations in the war against terrorism in recent decades. High-altitude mountainous terrains pose unique combinations of physiological stressors stemming from hypoxic exposure, low temperature, unstable weather, and rugged terrain (Lechner et al., 2018). A possible countermeasure to performance decline in this situation is the administration of exogenous ketone monoester (KME) as it rapidly alters metabolism via lowering circulating glucose levels and possibly lactate concentrations, while providing extrahepatic tissues and the brain with an alternate fuel substrate in the form of R-beta-hydroxybutyrate (βHB) (Evans et al., 2022).

Ketosis refers to the presence of elevated levels of ketone bodies in the blood (>0.5 mM βHB). In human physiology, “ketone bodies” refer to the metabolites βHB, acetoacetate (AcAc), and acetone; these small molecules are produced naturally in the liver by use of lipids, under conditions of low carbohydrate availability. Crucially, ketone bodies act as an alternative substrate to sustain cerebral metabolism when glucose concentration is less, accounting for up to 80% of brain energy metabolism under conditions of prolonged starvation (Owen et al., 1967). Glucose storage is relatively limited, i.e., capable of storing enough energy to maintain brain function for 24–48 h. In contrast, the energy reservoir represented by stored lipids is vast. However, lipids cannot be oxidized and transported to the brain in sufficient quantities to sustain brain function (Schönfeld and Reiser, 2013). The conversion of fat into ketones makes this pool of energy accessible to the brain as ketone bodies can readily cross the blood–brain barrier. Beyond their role as a metabolic substrate, ketone metabolism offers further advantages in the brain and other organs. First, the mitochondrial oxidation of ketones is energetically advantageous, increasing the efficiency of an isolated perfused heart by 24% compared to the metabolism of glucose alone (Sato et al., 1995). Second, ketones can protect mitochondrial function as they can act as scavengers of potentially harmful reactive oxygen species (Haces et al., 2008). Furthermore, ketones are endogenous inhibitors of histone deacetylase (HDAC) enzymes (Shimazu et al., 2013) and can thus modulate the gene expression. The downstream effects of HDAC inhibition are diverse, but one pertinent effect is the upregulation in the expression of brain-derived neurotrophic factor (BDNF) (Sleiman et al., 2016). Finally, ketones may have a clinically relevant systemic and neurological anti-inflammatory effect through the inhibition of the NLRP3 inflammasome (Yamanashi et al., 2020; Youm et al., 2015). These combined effects make ketones an attractive, natural strategy to sustain and protect the body and brain.

Nutritional (diet-induced) ketosis is typically induced by fasting or by following a ketogenic diet (i.e., a diet consisting of very low carbohydrate, low–moderate protein, and a high fat:macronutrient ratio). The time taken to reach a state of ketosis depends on many factors (i.e., habitual diet and exercise status). Generally, ketone levels exceed 0.5 mM after 24–48 h of fasting and can take up to 10 days to reach a “physiological” plateau at 6–8 mM (Cahill, 2006; Krebs, 1966). Blood ketone levels are dependent on adherence and time of the diet, with reported values ranging from 0.8 mM up to 4 mM (Edwards et al., 2011; Gilbert et al., 2000). However, the use of the ketogenic diet or fasting to achieve ketosis is widely considered to be impractical in operational settings due to the difficulty in sustainability and unwanted side effects, such as cramping or even impaired mitochondrial efficiency and cognitive function (Edwards et al., 2011). Consumption of exogenous KME may provide some of the benefits of ketone metabolism rapidly and without the requirement for fasting or a restrictive ketogenic diet.

For example, consumption of a KME, such as R,1-3 beta-hydroxybutyrate and R,1-3 butanediol, can deliver rapid (<30 min) and sustained (∼4 h) ketosis in a predictable, dose-dependent manner (Clarke et al., 2012; Shivva et al., 2016; Stubbs et al., 2017). This KME has undergone extensive safety testing to demonstrate its generally recognized as safe (GRAS) status, as recognized by the US Food and Drug Administration and has been studied in healthy adults at rest (Clarke et al., 2012; Shivva et al., 2016; Soto-Mota et al., 2019; Stubbs et al., 2017), during and after exercise (Cox et al., 2016; Evans and Egan, 2018; Vandoorne et al., 2017), and in clinical case studies (Bleeker et al., 2020). The KME undergoes hydrolysis in the gut to form βHB and butanediol; the latter undergoes hepatic conversion to βHB and, thus, elevates βHB levels without the fat, salt, or acid load accompanying other consumable ketone sources. Blood ketone levels can be elevated to >4 mM without depletion of carbohydrate stores, even with a carbohydrate-rich meal (Stubbs et al., 2017).

Prior studies provide preliminary evidence suggesting that KME might be effective in supporting mountaineering operations. First, there is evidence that exogenous ketone ingestion can mitigate the decline in cognitive performance after intense exercising (Evans and Egan, 2018; Prins et al., 2021). In laboratory studies, exogenous ketone ingestion has also been shown to enhance resiliency against decreases in oxygen saturation under hypoxic conditions (Poffé et al., 2021) and to be protective of cognitive performance against hypoxia (Prins et al., 2021) and metabolic stress, two prominent challenges in mountaineering operations and training.

Despite these promising recent findings, to our knowledge, there are no studies examining the effect of KME in real-life high-altitude mountaineering training. Thus, we conducted a double-blind placebo (PLA)-controlled study examining the efficacy of KME supplementation on the mitigation of cognitive performance decrease during and after rigorous mountaineering training among elite operators. We studied volunteers from the US military’s premier mountaineering training course that trains special operations personnel. We collected established neurocognitive functioning measures at the basecamp level (7,500 ft) before and after each of 2 days of mountaineering training, as well as at near-peak altitudes during these training days (11,000 ft and 14,000 ft).

## 2 Methods

### 2.1 Participants

Thirty-four participants were recruited from the US Army 10th Special Forces Group Special Operations Advanced Mountaineering School (SOAMS). The SOAMS is based out of Fort Carson, Colorado, and trains special operations personnel to navigate through and survive in mountainous terrain during military operations. Within the 7-week course, we studied participants at two stages: first, during the initial fitness evaluation event, called the Incline Fitness Test (IFT: see below), and second, during 2 consecutive, tactical mountain operations training days (two courses over 2 days) during the SOAMS Alpine Week, which was week 5 of the 7-week course. We conducted our assessments before, during, and after these 2 alpine training days. Table 1 shows the demographic characteristics of the enrolled participants. Four participants withdrew their participation before study day 1 due to logistical constraints or injury. Two additional participants withdrew after day 1 due to injury. The final numbers of participants were thus N = 30 for day 1 and N = 28 for day 2. One participant reported practicing a ketogenic diet. However, his blood ketone level during the baseline testing was 0.1 mmol/L, which is well below the general threshold for nutritional ketosis of 0.5 mmol/L and equivalent to other participants who did not report practicing a ketogenic diet. Thus, we treated this participant the same as others in the analyses.

**TABLE 1:** Participant demographic information (N = 34).

Characteristic	Mean (SD) or count (%)
Age	31.6 (4.24)
Height (cm)	179 (6.44)
Weight (kg)	85.0 (6.62)
Race	
White	30 (90.9%)
Other	3 (9.1%)
Education	
1–3 years after high school*	19 (57.6%)
College graduate	6 (18.2%)
High school graduate	8 (24.2%)

*Some college, associate’s degree, or technical school

Each participant provided electronic consent to participate after written and verbal explanations of the procedures in accordance with the protocol approved by the Florida Institute for Human & Machine Cognition Institutional Review Board and Office of Human Research Oversight, US Army Medical Research and Development Command. The protocol followed DoD Instruction 3216.02, “Protection of Human Subjects and Adherence to Ethical Standards in DoD-Conducted and Supported Research.” A waiver of documentation of informed consent (i.e., signatures) was warranted as per the requirements outlined in 32 CFR 219.117 (c). The current study qualified for this waiver as it presented only a minimal risk of harm to the participants and did not involve a procedure that required written consent outside the context of the research.

### 2.2 Study design, ketone monoester, and placebo drink

We used a double-blind, placebo–controlled, randomized crossover design examining the effect of KME vs. PLA on cognitive performance consequent to intensive training at high altitude. We used a commercially available KME (deltaG Tactical, TdeltaS Global, Inc.). The final active drink bottle contained 25 mL (26.7 g) of KME diluted in 25 mL of water, with 50 mL in total in a 1:1 ratio. The PLA drink was prepared to mimic both the bitter taste and viscosity of the active drink by mixing a taste-bittering agent (denatonium benzoate), a thickener (arrow root powder), and water, also apportioned to 50 mL to match the volume in the active KME bottle. The active drink and placebo were prepared by staff not involved in data collection, and each bottle was labeled as “A” or “B” such that neither the data collectors nor participants knew whether A or B contained the active KME.

**FIGURE 1:**
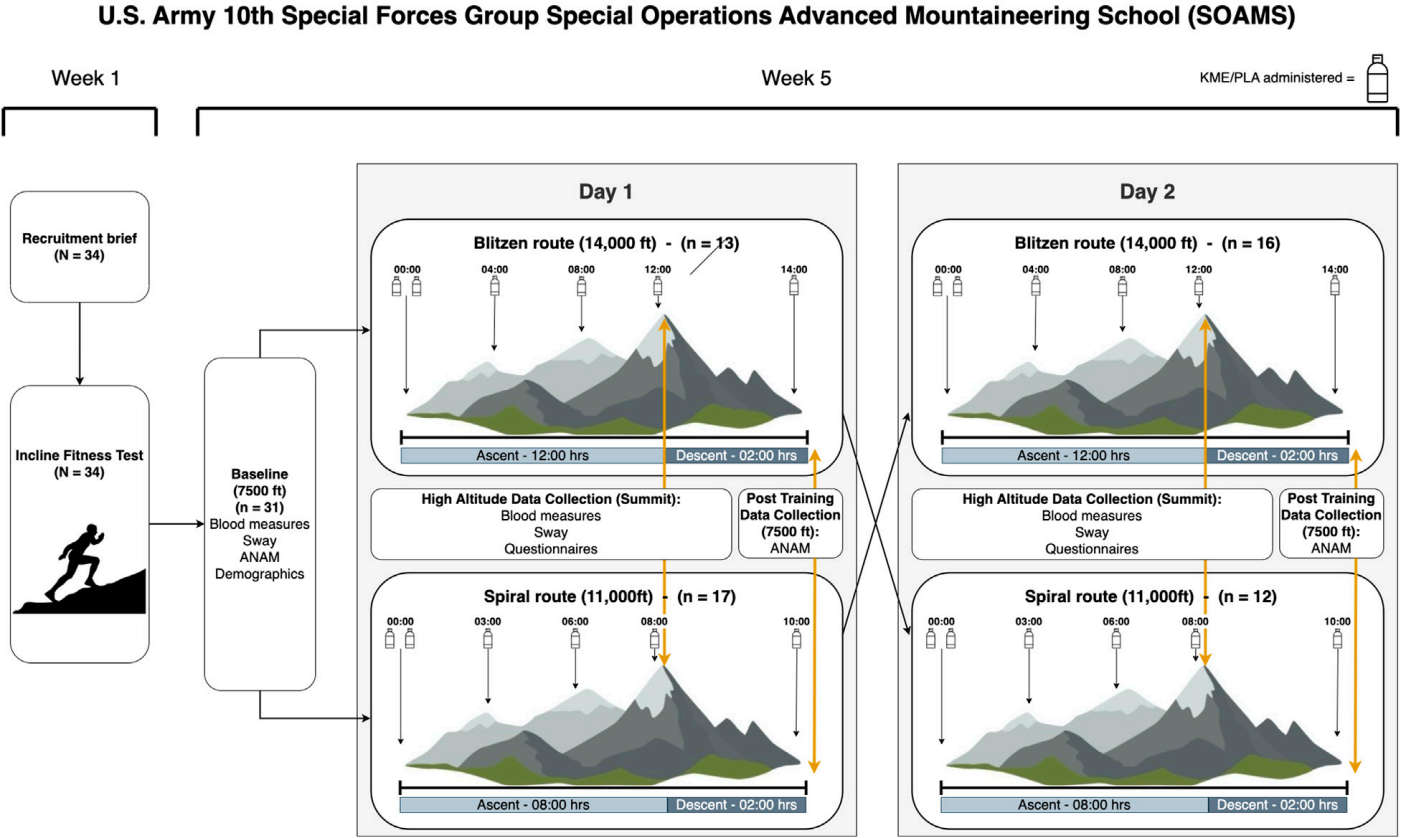
Schematic illustration of the data collection schedule.

Figure 1 shows a schematic illustration of the data collection schedule. On each of the 2 testing days, each participant consumed six 50-mL bottles of either A or B as follows: bottles #1 and #2 at the trailhead prior to beginning of mountaineering training, #3 at 25% completion of the training day, #4 at 50% completion of the training day, #5 at approximately 75% completion of the training day (the time at which participants and researchers met near peak altitude for on-mountain data collection), and #6 just prior to arriving at the 7,500 ft basecamp for the final data collection of the day.

### 2.3 Incline fitness test

The incline fitness test is part of the initial series of fitness and skills tests that must be completed to be admitted to the SOAMS course. The requirement is to complete the Manitou Incline in less than 45 min. Students are incentivized to perform the task as quickly as possible since not all students have a guaranteed slot in the course; their admission is based on their performance in the preliminary fitness and skill tests.

The Incline consists of 2,744 steps, covering 0.9 miles and 2,020 ft/615 m of elevation gain. The average grade is 41%, with the steepest grade being 68%. The base elevation is 6,530 ft/2,012 m, and the summit elevation is 8,550 ft/2,606 m.

Students wore Garmin Fenix 7X Pro Solar watches during the test to collect speed and heart rate data. Once students reached the peak of the incline, their finishing times were recorded, and they were administered the Borg Rating of Perceived Exertion scale (Williams, 2017) and the Samn–Perelli Fatigue Scale (Petrilli et al., 2006). These tests were done to establish a baseline for pace, heart rate, effort, and fatigue for comparison to the 2 mountaineering days with KME vs. PLA drinks.

### 2.4 Baseline data collection (7,500 ft/2,286 m)

One day before the mountaineering training days, participants gathered at a cabin at 7,500 ft for the baseline data collection. The baseline data collection consisted of neurocognitive functioning tests (Automated Neuropsychological Assessment Metrics version 4 [ANAM-4] and SWAY), questionnaires (medical history, exercise history, demographic, alcohol and tobacco use, and diet history including whether they practiced a ketogenic diet), and blood glucose and ketone measurements. Detailed descriptions of these and other measurements are provided below.

### 2.5 High-altitude data collection

On each of the 2 days of high-altitude data collection, the participants completed a neurocognitive functioning test (SWAY); questionnaires on perceived exertion (Borg scale), fatigue (Samn–Perelli Fatigue Scale), and gastrointestinal symptoms (GI symptom questionnaire); as well as blood glucose and ketone measurements at a test site near peak altitude each day. These sites were selected to have enough space on a relatively flat terrain but still at or close to the highest altitude for that course (see below) just prior to beginning the day’s final descent.

### 2.6 Summary of the two mountaineering training routes studied during the SOAMS Alpine Week

#### 2.6.1 Blitzen route (study data collection at 13,500 ft/4,118 m)

The Blitzen route totaled 14 h of continuous training. This included over 10 h of training above 11,000 ft (tree line), reaching a peak of 13,627 ft. On this route, participants completed 305 m/1,000 ft of 5th class climbing (steep, highly technical, roped climbing), traversed 3.2 km/2 miles of 4th class terrain, carried 9.07 kg/20 l b of gear, completed several rappels, and worked in teams of 3 on belay, short roping, and short pitching techniques. Across the training day, the total distance covered was ∼17.54 km/10.9 miles.

#### 2.6.2 Spiral route (study data collection at 11,000 ft/3,352 m)

The spiral route totaled 10 h of continuous training. This included over 6 h of training above 11,000 ft (tree line), reaching a peak of 12,460 ft. On this route, participants completed 335 m/1,100 ft of 5^th^ class climbing (steep, highly technical, roped climbing) and several long rappels. Across the training day, the total distance covered was ∼14.65 km/9.1 miles.

### 2.7 Post-training data collection (7,500 ft/2,286 m)

Participants completed a neurocognitive functioning test (ANAM-4) at the basecamp cabin or at their nearby hotel after they completed each day of the mountaineering training and descended from the high-altitude data collection sites.

### 2.8 Data

#### 2.8.1 Garmin Fenix 7X Pro Solar

Garmin Fenix 7X Pro Solar (Garmin Ltd., Schaffhausen, Switzerland) is a multi-functional sport watch equipped with a PPG, GPS, electronic compass, altimeter, barometer, temperature sensor, and SpO_2_. Through a combination of these sensor inputs, Fenix 7X Pro Solar computes advanced performance metrics. Garmin watches have been validated against gold standard measures for accuracy in several studies, and they are generally considered one of the most reliable commercial-off-the-shelf (COTS) devices for HR measurement (Fuller et al., 2020). Participants wore the device during the Incline Fitness Test, High-Altitude Training days, and for approximately 1 month in between these data collection events. We used the continuous heart rate and activity data, such as elevation, distance traveled, and their timestamp, as the measures of interest.

#### 2.8.2 Oura Ring Gen2

The Oura Ring is a lightweight (15 g) multisensory device (Oura Health Oy, Oulu, Finland) that tracks sleep metrics (e.g., duration, timing, heart rate, and heart rate variability). It uses infrared light and a photosensor to gather beat-by-beat blood volume pulse architecture. The ring is also equipped with an accelerometer that tracks body movement amplitude and intensity, as well as a negative temperature coefficient thermistor that tracks skin temperature with high resolution. The sleep and heart rate measurements of the Oura Ring have previously been validated against research-grade actigraphy and polysomnography (Chee et al., 2021). Participants wore the ring during sleep from the recruitment brief until the conclusion of the study. We used five sleep metrics from the night before (lowest heart rate, average heart rate, RMSSD [i.e., heart rate variability and HRV], average respiratory rate, and total sleep duration) as the variables of interest. We also computed the 3-day average of these five metrics given the findings that not only is the sleep quality from the night before but also the totality of the sleep quality of multiple days important in assessing cognitive readiness. We also included the Oura readiness score and sleep score. They are composite scores given in their commercial smartphone application. Although the formulas used to compute these metrics are proprietary, and, as such, they are not ideal as research variables, we decided to include them as potentially useful descriptive metrics.

#### 2.8.3 Precision Xtra

Precision Xtra is a device for measuring blood glucose and ketone levels (Abbott Laboratories, Chicago, IL), most commonly used for monitoring blood glucose levels of diabetic patients. It requires only 1.5 μL of blood and provides the result (mg/dL) in 5 (glucose)–10 (ketone) s. The researchers administered the test by obtaining a drop of blood from the participants’ point finger using a finger prick and applying the blood to the test strips for evaluation of glucose and ketone levels.

#### 2.8.4 Automated Neuropsychological Assessment Metrics version 4

Automated Neuropsychological Assessment Metrics version 4 (ANAM-4) is a test of neurocognitive functioning designed to detect cognitive abnormality, such as traumatic brain injuries. Participants completed a set of 10 tasks from the core battery designed to measure various aspects of neurocognitive functioning. The specific tests that we employed were code substitution—learning and code substitution—delayed (CDS and CDD: measure of sustained attention and working memory), go/no-go (GNG: measure of response inhibition), matching to sample (M2S: spatial processing and visual working memory), mathematical processing (MTH: measure of basic computational skills, concentration, and working memory), procedural reaction time (PRO: information processing speed, visuomotor reaction time, and attention), spatial processing (SPD: visual skills and mental rotation), simple reaction time and simple reaction time—repeat (SRT and SR2: reaction time), and memory search (ST6: verbal short-term memory). The median reaction time of correct trials for each test was taken as the variable of interest.

#### 2.8.5 SWAY

SWAY is an accelerometer-based test of balance and cognitive performance originally developed for concussion testing. We administered the simple reaction time and impulse control (measure of response inhibition much like ANAM-4 go/no-go) tests (VanRavenhorst-Bell et al., 2021) as a portable cognitive test at the high-altitude testing sites.

#### 2.8.6 Questionnaires

##### 2.8.6.1 Personal history

To characterize a participant’s health and lifestyle, we administered the following questionnaires at the baseline: (1) medical history; (2) medication history; (3) exercise history; (4) alcohol/tobacco history; (5) diet and supplement history; (6) demographics; and (7) self-health assessment.

##### 2.8.6.2 RPE scale

We used the Borg RPE scale to measure the subjective perception of physical demands during the mountaineering training. The Borg scale is a 15-point scale (6–20) ranging from 6, “no exertion,” to 20, “maximal exertion” (Williams, 2017).

##### 2.8.6.3 Samn–Perelli Fatigue Scale

We used the Samn–Perelli Fatigue Scale to measure subjective fatigue, which was originally developed during aviation research (Petrilli et al., 2006). The Samn–Perelli Fatigue Scale is a 7-point Likert scale ranging from 1, “fully alert, wide awake,” to 7, “completely exhausted, unable to function efficiently.”

##### 2.8.6.4 Gastrointestinal symptom questionnaire

The gastrointestinal (GI) symptom questionnaire was originally developed at the IHMC. It measures the presence and severity of four symptoms under three categories (upper abdominal problems: heartburn, bloating, nausea, and vomiting; lower abdominal problems: intestinal cramps, abdominal pain, flatulence, and diarrhea; and systemic problems: dizziness, headache, muscle cramp, and urge to urinate) on a 9-point scale (0–8) ranging from 0, “none,” to 4, “moderate,” and 8, “unbearable.”

### 2.9 Data management and statistical analysis

#### 2.9.1 Data collection and analysis tools

All history questionnaires were collected via the Smartabase (product of Teamworks, Durham, North Carolina, United States) database management system. Participants completed the questionnaires via their participant accounts, and the data were reviewed for completion by members of the research team. Data of Garmin and Oura wearables were synchronized from their cloud platform to Smartabase and extracted from there via an application programming interface. All statistical analyses were performed in R. The data pipeline for processing and analysis was obtained using the Make-like pipeline tools package targets and the supporting tarchetypes package. Data visualization and quality analysis were completed using internally developed apps (Shiny).

#### 2.9.2 QA-QC and outlier detection

Datasets were investigated for outliers and reviewed for quality assurance–quality control (QA-QC). Values determined to be outliers and invalid by subject matter experts were removed from analysis. Documentation of all values removed is provided in the code base. First, within a variable, if the value was ≥3.6 standard deviations above or below the mean, the value was flagged as an outlier and removed. We also used minimum covariance determinant (MCD) to calculate robust Mahalanobis distances (univariate and multivariate) on key outcomes. *p*-values were calculated from a chi-squared distribution, and an alpha value of 0.001 was used to determine whether a value was an outlier. Any values flagged as outliers were reviewed by subject matter experts to determine whether their validity.

#### 2.9.3 Modeling of effects

Due to the nature of the training course, we could not have participants consume both KME and PLA on both mountain routes. This led to two potential sets of factors that could impact dependent variables: effects of both time (baseline, day 1, and day 2) and route (Spiral vs. Blitzen). To determine which parameters best explained the outcome, we produced two models and compared them for fit: 1) treatment and time point as fixed effects and 2) treatment and route as fixed effects. We fit both mixed-effects models using the maximum likelihood estimate of the model and compared the two using the Akaike information criterion (AIC). Following the convention in interpreting the AIC (Burnham and Anderson, 2004), we considered an AIC difference of 2 or greater as meaningful. For the 12 cognitive test comparisons (ANAM-4 and SWAY), 7 tests had a meaningfully better fit for the time model, 2 tests had a meaningfully better fit for the route model, and 3 tests had comparable fit (i.e., AIC difference < 2). For subjective questionnaires on physical exertion and fatigue (Borg RPE and Samn–Perelli), both comparisons had a meaningfully better fit for the route model. For blood glucose and ketone measurements, blood glucose had a meaningfully better fit for the time model, whereas blood ketone had a meaningfully better fit for the route model. Based on these comparisons, we report the time models for the cognitive tests, route models for the subjective questionnaires, and both models for blood glucose and ketone measures in the main text. However, we also report the results of the unreported models in Supplementary Material (cognitive test route models: Supplementary Figures S5, S6; Supplementary Table S2; subjective questionnaire time models: Supplementary Figure S7; Supplementary Table S3) for completeness.

We created the final model with the same random factor of participant (random intercept) as the model comparison step but re-fit the model using restricted maximum likelihood (REML) estimation. Formal hypotheses, regarding the main effects of treatment (KME or PLA) and time point (baseline, day 1, and day 2) or route (Blitzen or Spiral), as well as the treatment × time point or treatment × route interaction, were tested using a mixed-effects ANOVA (the degrees of freedom used to calculate the F-statistic were estimated from the REML mixed-effects model using the Satterthwaite method). Alpha was set at 0.05. All models were inspected visually using residual plots.

## 3 Results

### 3.1 Oura Ring sleep data

**FIGURE 2:**
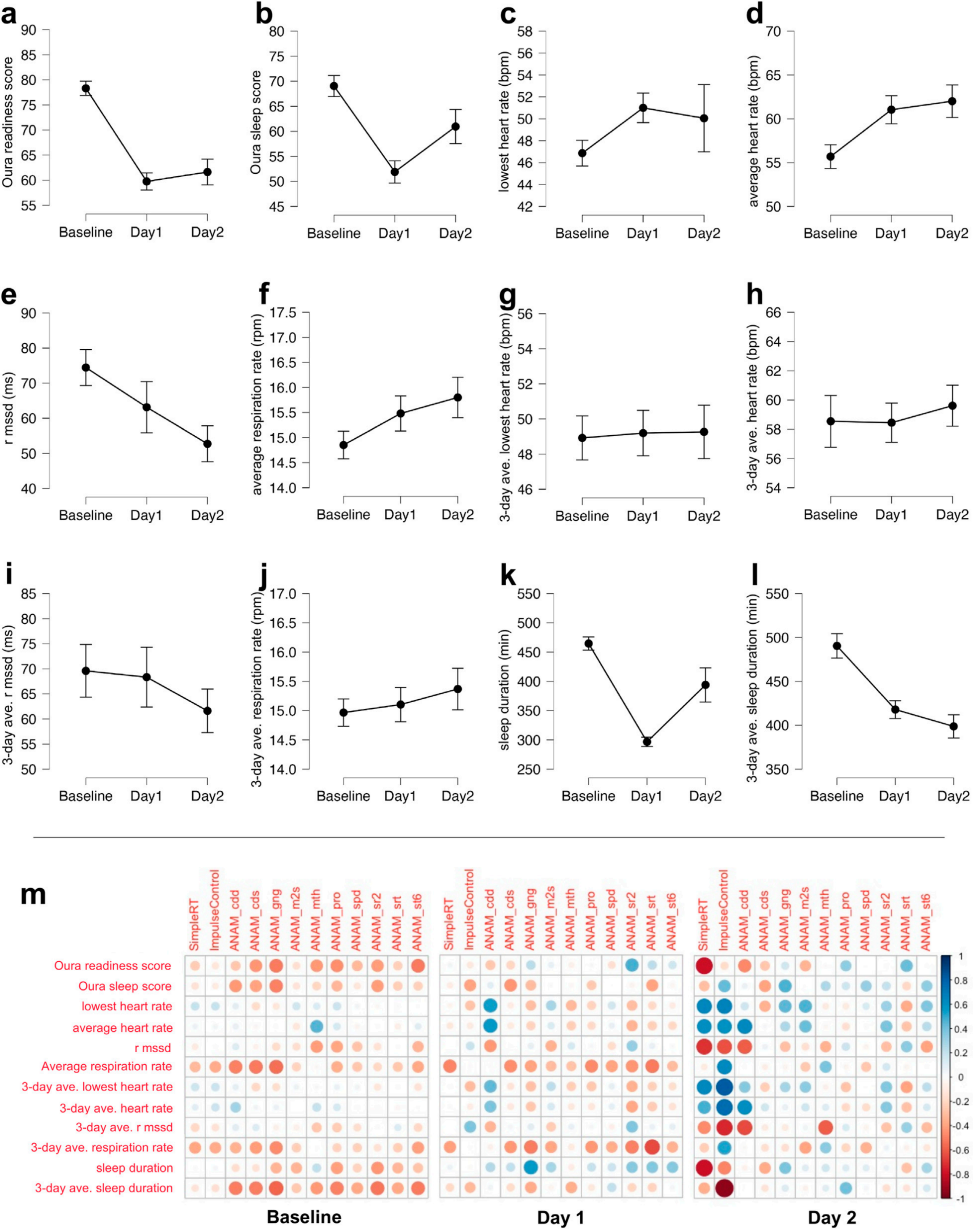
(a–l) Sleep and readiness metrics derived from the Oura Ring plotted by the baseline testing day, alpine training day 1, and alpine training day 2. The effort bars denote the standard error: **(A)** Oura readiness score; **(B)** Oura sleep score; **(C)** lowest heart rate during sleep from the night before in beats per minute (BPM); **(D)** average heart rate during sleep from the night before in BPM; **(E)** RMSSD during sleep from the night before; **(F)** average respiratory rate during sleep from the night before in respiration per minute (RPM); **(G)** 3-day average of the lowest heart rate during sleep from the night before in BPM; **(H)** 3-day average of the average heart rate during sleep from the night before in BPM; **(I)** 3-day average of RMSSD during sleep from the night before; **(J)** 3-day average of the average respiratory rate during sleep from the night before in RPM; **(K)** sleep duration from the night before in minutes; **(L)** 3-day average of sleep duration from the night before in minutes; **(M)** partial correlation matrices between the sleep metrics that were derived from the Oura Ring and cognitive performance at the baseline testing (left panel), as well as at the high altitude (11,000 or 14,000 ft: simpleRT and ImpulseControl in SWAY) and post-training at the basecamp level (7,500 ft: ANAM variables) on alpine days 1 (middle panel) and 2 (right panel). *cdd*, code substitution—delayed; *cds*, code substitution learning; *gng*, go/no-go; *m2s*, matching to sample; *mth*, mathematical processing; *pro*, procedural reaction time; *spd*, spatial processing; *sr2*, simple reaction time second time; *srt*, simple reaction time first time; *st6*, memory search. All ANAM variables are the median reaction time for correct trials from the given test.

Figure 2 shows the sleep metrics derived from the Oura Ring plotted by time point (baseline, day 1, and day 2) and the partial correlation matrices between sleep metrics and cognitive performance for the baseline testing, at high altitude (11,000 or 14,000 ft: SimpleRT and ImpluseControl in SWAY) and post-training at the basecamp level (7,500 ft: ANAM variables).

#### 3.1.1 Sleep metrics across the testing time point

Supplementary Table S1 shows the full results of one-way ANOVA examining the effect of time point on the sleep and readiness metrics. Pertinent to the main interest of the current investigation, participants’ sleep duration (in minutes) decreased significantly during alpine training days (day 1: *M* = 296.70, *SD* = 35.20; day 2: *M* = 394.00, *SD* = 130.56) compared to the night before baseline testing (*M* = 464.64, *SD* = 53.00), *F*(2,59) = 21.53, *p* < .001, which was also reflected in the 3-day average of the sleep duration decreasing significantly during alpine training days (day 1: *M* = 417.91, *SD* = 46.31; day 2: *M* = 398.76, *SD* = 58.97) compared to the night before baseline testing (*M* = 490.39, *SD* = 65.28), *F*(2,59) = 15.06, *p* < .001. This reduction in sleep duration coincided with significant decreases in various sleep and readiness metrics, such as readiness score (baseline: *M* = 78.32, *SD* = 6.64; day 1: *M* = 59.75, *SD* = 7.66; day 2: *M* = 61.63, *SD* = 11.20), *F*(2,59) = 29.80, *p* < .001; sleep score (baseline: *M* = 69.01, *SD* = 9.81; day 1: *M* = 51.90, *SD* = 9.92; day 2: *M* = 60.95, *SD* = 15.17), *F*(2,59) = 11.00, *p* < .001; average heart rate during sleep (baseline: *M* = 55.69, *SD* = 6.36; day 1: *M* = 61.04, *SD* = 7.19; day 2: *M* = 62.01, *SD* = 8.32), *F*(2,59) = 4.62, *p* = .014; and RMSSD (i.e., HRV) during sleep (baseline: *M* = 74.46, *SD* = 24.12; day 1: *M* = 63.15, *SD* = 32.66; day 2: *M* = 52.74, *SD* = 22.37), *F*(2,59) = 3.38, *p* = .041.

#### 3.1.2 Baseline cognitive performance and sleep metrics

The Oura readiness score was inversely correlated with ANAM go/no-go, *r*(19) = −.50, *p* = .02; ANAM procedural reaction time, *r*(19) = −.45, *p* = .04; and ANAM memory search, *r*(19) = −.51, *p* = .02. The Oura sleep score was inversely correlated with ANAM code substitution delayed, *r*(19) = −.54, *p* = .01; ANAM code substitution learning, *r*(19) = −.48, *p* = .03; and ANAM go/no-go, *r*(19) = −.50, *p* = .02. The average heart rate during sleep correlated with ANAM mathematical processing, *r*(19) = .43, *p* = .049. The average respiration rate from the night before was inversely correlated with ANAM code substitution learning, *r*(19) = −.48, *p* = .03 and ANAM go/no-go, *r*(19) = −.50, *p* = .02. The average respiration rate over the three previous nights was inversely correlated with ANAM go/no-go, *r*(19) = −.46, *p* = .04. The total sleep duration from the night before was inversely correlated with ANAM go/no-go, *r*(19) = −.45, *p* = .04. The average total sleep duration over the three previous nights was inversely correlated with ANAM go/no-go, *r*(19) = −.50, *p* = .02 and ANAM simple reaction time repeat, *r*(19) = −.52, *p* = .02.

#### 3.1.3 Alpine day-1 cognitive performance and sleep metrics

The lowest heart rate from the night before was correlated with ANAM code substitution delayed, *r*(12) = .56, *p* = .04. The average heart rate from the night before was correlated with ANAM code substitution delayed, *r*(12) = .56, *p* = .04. The average respiration rate from the night before was inversely correlated with SWAY simple reaction time, *r*(16) = −.48, *p* = .04. The average respiration rate over the three previous nights was inversely correlated with ANAM simple reaction time, *r*(13) = −.64, *p* = .01. The total sleep duration from the night before was correlated positively with ANAM go/no-go, *r*(12) = .57, *p* = .03.

#### 3.1.4 Alpine day-2 cognitive performance and sleep metrics

The lowest heart rate from the night before was correlated with ANAM matching to sample, *r*(16) = .47, *p* = .049. The average heart rate during sleep from the night before was correlated with code substitution delayed, *r*(16) = .63, *p* < .01. HRV from the night before was inversely correlated with code substitution delayed, *r*(15) = −.59, *p* = .01, and mathematical processing, *r*(16) = −.60, *p* < .01. Note that none of the apparent correlations between sleep metrics and SWAY variables (simple reaction time and impulse control) are statistically significant due to the low sample size (n = 5 for simple reaction time and n = 4 for impulse control) stemming from participant drop out and technical difficulties at the high altitude.

In general, nearly all significant correlations (except total sleep duration vs. ANAM go/no-go on day 1) are in the expected direction. Overall, these results suggest the autonomic tone and readiness gaged from the Oura Ring accounted for a meaningful portion of variability in cognitive performance. They also emphasize the importance of considering sleep metrics from multiple consecutive previous nights when relating sleep to cognitive performance.

### 3.2 Garmin activity data

#### 3.2.1 Incline fitness test

On average, across all N = 34 participants, the completion time for the IFT was 35.2 min (*SD* = 4.5, minimum = 28, maximum = 46.5) with an average RPE of 17 out of 20 (“very hard”), indicating that the IFT was a short but intense physical test. Due to issues with compliance and data QA-QC, Garmin data from the incline fitness test were available for analysis from only N = 23 participants. We extracted the test data from Garmin via Smartabase, from which we isolated the IFT data by filtering the data based on the break point at which elevation reported by the Garmin watch increased exponentially to determine the start of the test, and the end of the test was determined when each participant reached peak elevation during the activity window. Supplementary Figure S1 summarizes the data extracted.

One participant was removed from the analysis (P418) due to invalid data based on QA-QC from the elevation and heart rate raw data plots. Supplementary Figure S1 shows elevation data, and Supplementary Figure S2 shows raw heart rate data. To verify that the extraction of the data from the Garmin was representative of the test, we ran a correlation test between the total completion time, as determined by the Garmin, versus that recorded manually by the cadre. There was a strong correlation between the Garmin-sampled total time and recorded time (*R* ^2^ = 0.98, *p* < 0.001).

**FIGURE 3:**
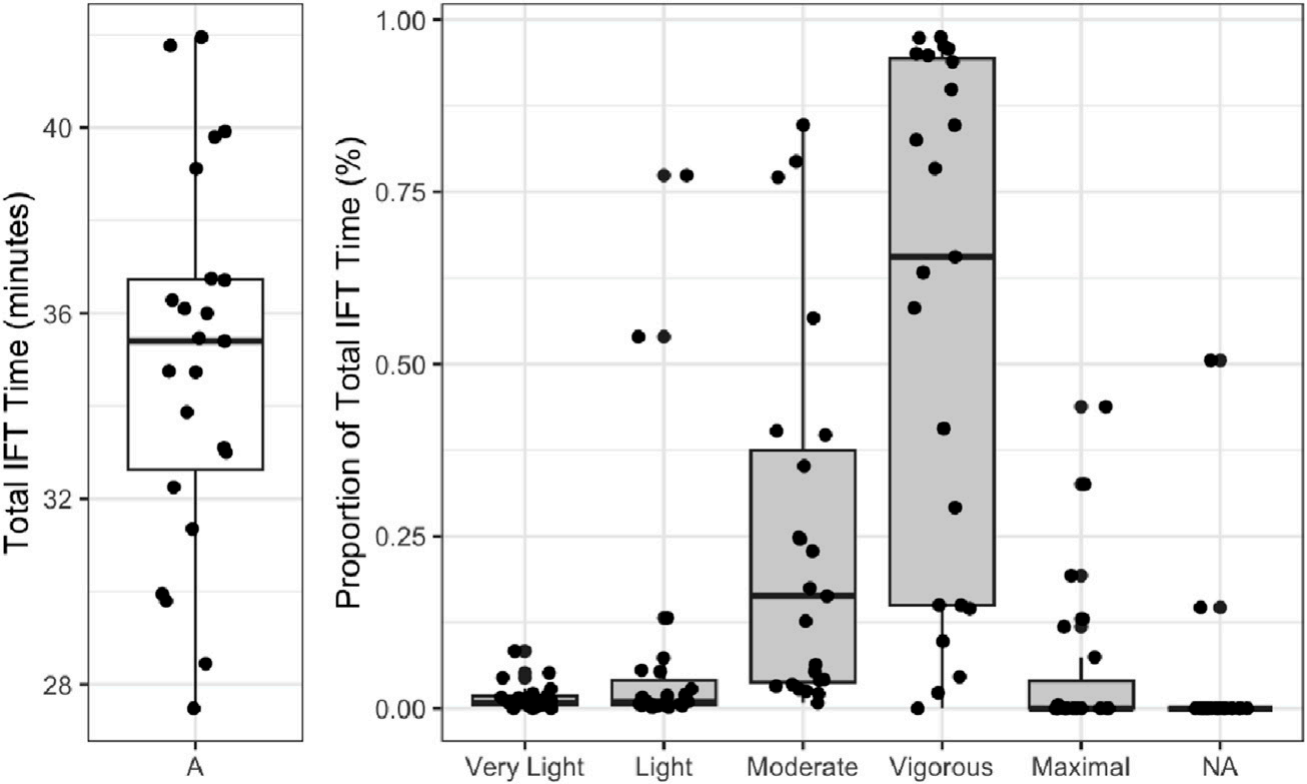
Proportion of the IFT time by the heart rate intensity zone. IFT, incline fitness test. Heart rate zones are defined by the American College of Sports Medicine defined zones and are calculated based on the percentage of age-predicted heart rate max. Very light <57% HR_max_; light, 57%–63%; moderate, 64%–76%; vigorous, 77%–95%; maximal, >95%.

Next, we summarized the heart rate zones during the incline fitness test. Figure 3 summarizes the total test time spent in each of the heart rate zone categories.

We conducted correlational analyses on each of these categories to explore potential intensity strategies for the IFT. There was no significant correlation between the total IFT time and any of the heart rate zones. This indicates that higher relative intensities are not related to ascending slower or faster on the IFT and may indicate that there are diverse strategies that can be used during the IFT.

#### 3.2.2 Alpine days

We extracted the ascent portion of the climb by extracting the start of the session up to the point at which the participant reached peak altitude. Three participants were removed due to having invalid elevation data. Another participant was removed due to a high volume of missing heart rate data. When removed, the data missingness for heart rate was reduced by 69%. Raw heart rate data tracings are shown in Supplementary Figures S3, S4.

**FIGURE 4:**
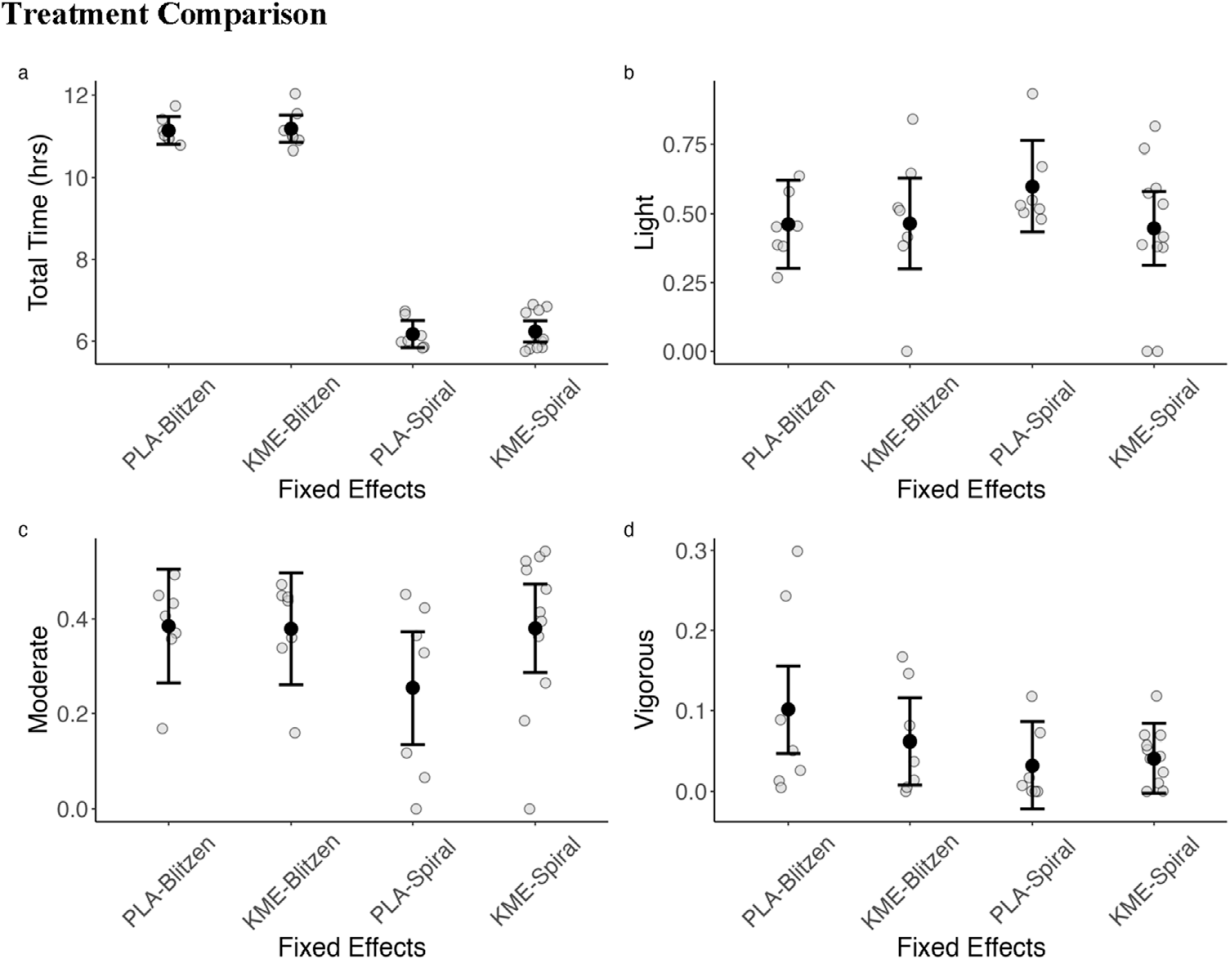
Summary of heart rate zones across each route by treatment (KME vs. PLA). **(A)** Total time in hours to complete the ascent portion of the routes. **(B)** Percentage of the total completion time spent in the light-intensity activity heart rate zone (57%–63% HR_max_). **(C)** Percentage of the total completion time spent in the moderate-intensity activity heart rate zone (64%–76%). **(D)** Percentage of the total completion time spent in the vigorous-intensity activity heart rate zone (95%). Heart rate zones are defined by the American College of Sports Medicine defined zones and are calculated based on the percentage of age-predicted maximum heart rate.

We fit mixed-effects models using treatment (KME vs. PLA) and route (Blitzen vs. Spiral) as fixed effects and participant as a random effect in the model (random intercept). Figure 4 shows the IQ range and median for each of the outcomes. There were no treatment-by-route interactions. There was abundant time to complete each route, even within a route. Therefore, we normalized the time spent in each category by calculating the percentage of total time that a participant spent in each given heart rate intensity zone. For light and moderate intensities, there were no significant treatment-by-route interactions, main effects of route, or main effects of treatment. For vigorous intensity, there was no interaction or main effect of treatment, but there was a main effect of route (*p* = 0.048).

### 3.3 Blood glucose and ketone levels

**FIGURE 5:**
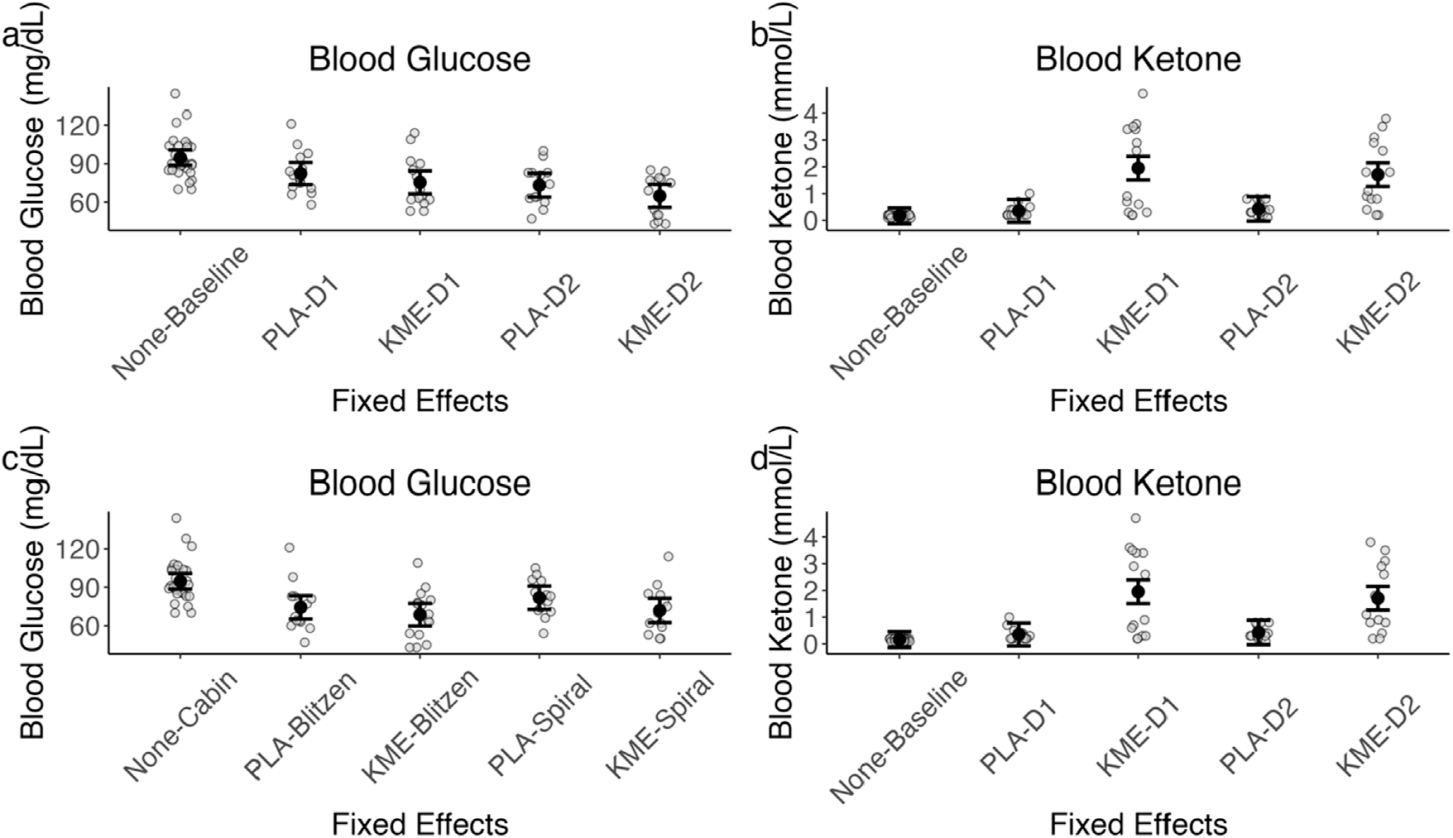
**(A)** Model-estimated means of blood glucose levels in mg/dL plotted by treatment and time point, where placebo = PLA, ketone monoester = KME, mountaineering day 1 = D1, and mountaineering day 2 = D2. **(B)** Model-estimated means of blood ketone levels in mmol/L plotted by treatment and time point. **(C)** Model-estimated means of blood glucose levels in mg/dL plotted by treatment and mountaineering route. **(D)** Model-estimated means of blood ketone levels in mmol/L plotted by treatment and mountaineering route. The error bars denote 95% CI.

Figure 5 plots the blood glucose and blood ketone levels, and Table 2 shows the test statistics. For the blood glucose level, with the treatment × time point model, there was a significant main effect of treatment, such that PLA had a greater blood glucose level at peak altitude than KME. The main effect of time point was also significant, indicating that the blood glucose level was greater on day 1 than on day 2. The interaction between these two variables was not significant. With the treatment × route model, there was also a significant main effect of treatment, such that PLA had a greater blood glucose level at peak altitude than KME.

**TABLE 2:** Blood glucose and ketone level ANOVA model results.

Model	Predictor	*df*_*Num*_	*df*_*Den*_	*Sum sq*	*Mean sq*	*F*	*p*
Blood glucose level	Treatment	2	56.81	10,415.79	5,207.89	18.83	<.001***
	Time	1	56.78	1,347.30	1,347.30	4.87	0.03*
	Treatment × time	1	58.95	7.12	7.12	0.03	0.87
Blood ketone level	Treatment	2	81.76	41.35	20.67	29.98	<.001***
	Time	1	81.76	0.096	0.096	0.14	0.71
	Treatment × time	1	81.76	0.36	0.36	0.52	0.47
Blood glucose level	Treatment	2	82	5,363.27	2,681.63	9.10	<.001***
	Route	1	82	408.94	408.94	1.39	0.24
	Treatment × route	1	82	60.91	60.91	0.21	0.65
Blood ketone	Treatment	2	57.23	35.01	17.51	26.99	<.001***
	Route	1	57.32	2.42	2.42	3.72	0.058^†^
	Treatment × route	1	58.51	0.61	0.61	0.93	0.34

^†^ *p <* .10, *p < .05*,* and ***p < .001.

For the blood ketone level, with the treatment × time point model, there was a significant main effect of treatment, such that the KME condition had a significantly greater blood ketone level at peak altitude than PLA. With the treatment × route model, there was a significant main effect of treatment, such that the KME condition had a significantly greater blood ketone level at peak altitude than PLA. The main effect of route was marginally significant, suggesting that blood ketone levels were greater after the Blitzen route than those after the Spiral route. The interaction between these two variables was not significant.

### 3.4 Borg rating of perceived exertion and Samn–Perelli Fatigue Scale

**FIGURE 6:**
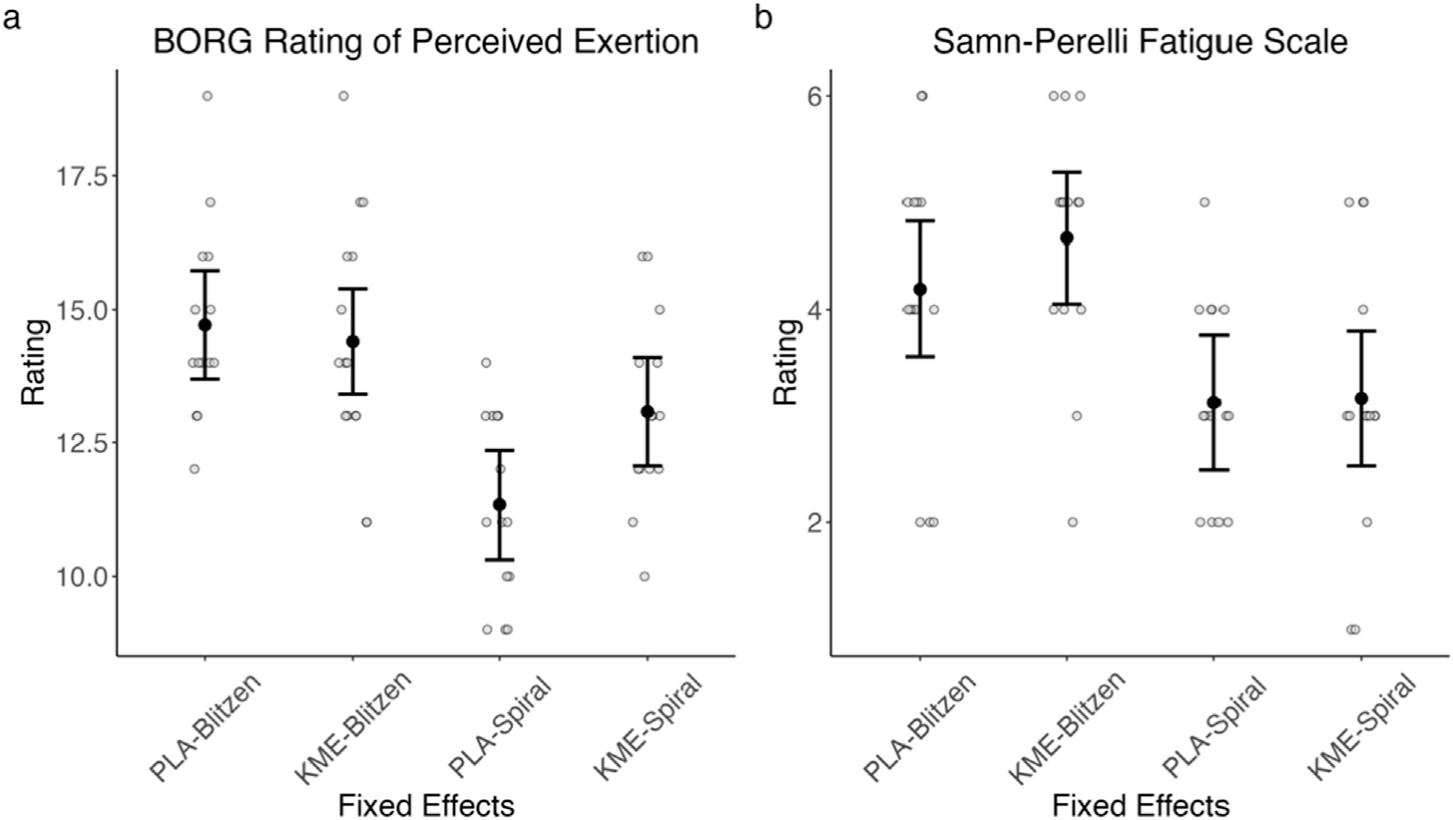
Model-estimated means of Borg RPE **(A)** and Samn–Perelli Fatigue Scale **(B)** ratings plotted by the treatment condition and the route, where PLA = placebo and KME = ketone monoester. The error bars denote 95% CI.

Figure 6 plots the Borg RPE and Samn–Perelli Fatigue Scale rating collected near peak altitude on each of the two training days, and Table 3 shows the test statistics. For the Borg RPE, with the treatment × route model, there was a marginally significant main effect of treatment, such that KME appeared to yield a higher RPE at peak altitude, specifically on the Spiral route, which had a shorter ascent duration and a lesser peak altitude than the Blitzen route. The main effect of route was significant, indicating that RPE was greater during the Blitzen route ascent than the Spiral route ascent. The interaction between these two variables was marginally significant. *Post hoc t*-tests showed significant or marginally significant differences in RPE between placebo-Blitzen and placebo-Spiral (t[47.95] = 4.71, *p* < 0.001), placebo-Blitzen and KME-Spiral (t[27.87] = 2.87, *p* = 0.01), KME-Blitzen and placebo-Spiral (t[26.96] = 5.53, *p* < 0.001), KME-Blitzen and KME-Spiral (t[47.33] = 1.87, *p* = 0.07), and placebo-Spiral and KME-Spiral (t[47.95] = −2.44, *p* = 0.02). No difference was noted in RPE between placebo-Blitzen and KME-Blitzen (t[47.33] = 0.44, *p* = 0.66).

**TABLE 3:** Borg Rating of Perceived Exertion scale and Samn–Perelli Fatigue Scale ANOVA model results.

Model	Predictor	*df*_*Num*_	*df*_*Den*_	*Sum sq*	*Mean sq*	*F*	*p*
Perceived exertion	Treatment	1	27.42	7.19	7.19	3.27	0.08^†^
	Route	1	27.42	77.03	77.03	34.98	<.001***
	Treatment × route	1	28.61	6.69	6.69	3.04	0.09
Perceived fatigue	Treatment	1	26.37	0.92	0.92	1.04	0.32
	Route	1	26.37	23.02	23.02	25.87	<.001***
	Treatment × route	1	27.48	0.32	0.32	0.36	0.55

^†^ *p < .*10 and ***p < .001.

For the Samn–Perelli Fatigue Scale rating, with the treatment × route model, there was no main effect of treatment. The main effect of route was significant, indicating that the level of subjective fatigue was greater after the Blitzen route ascent than after the Spiral route ascent. There was no significant interaction between these two variables.

### 3.5 ANAM-4

**FIGURE 7:**
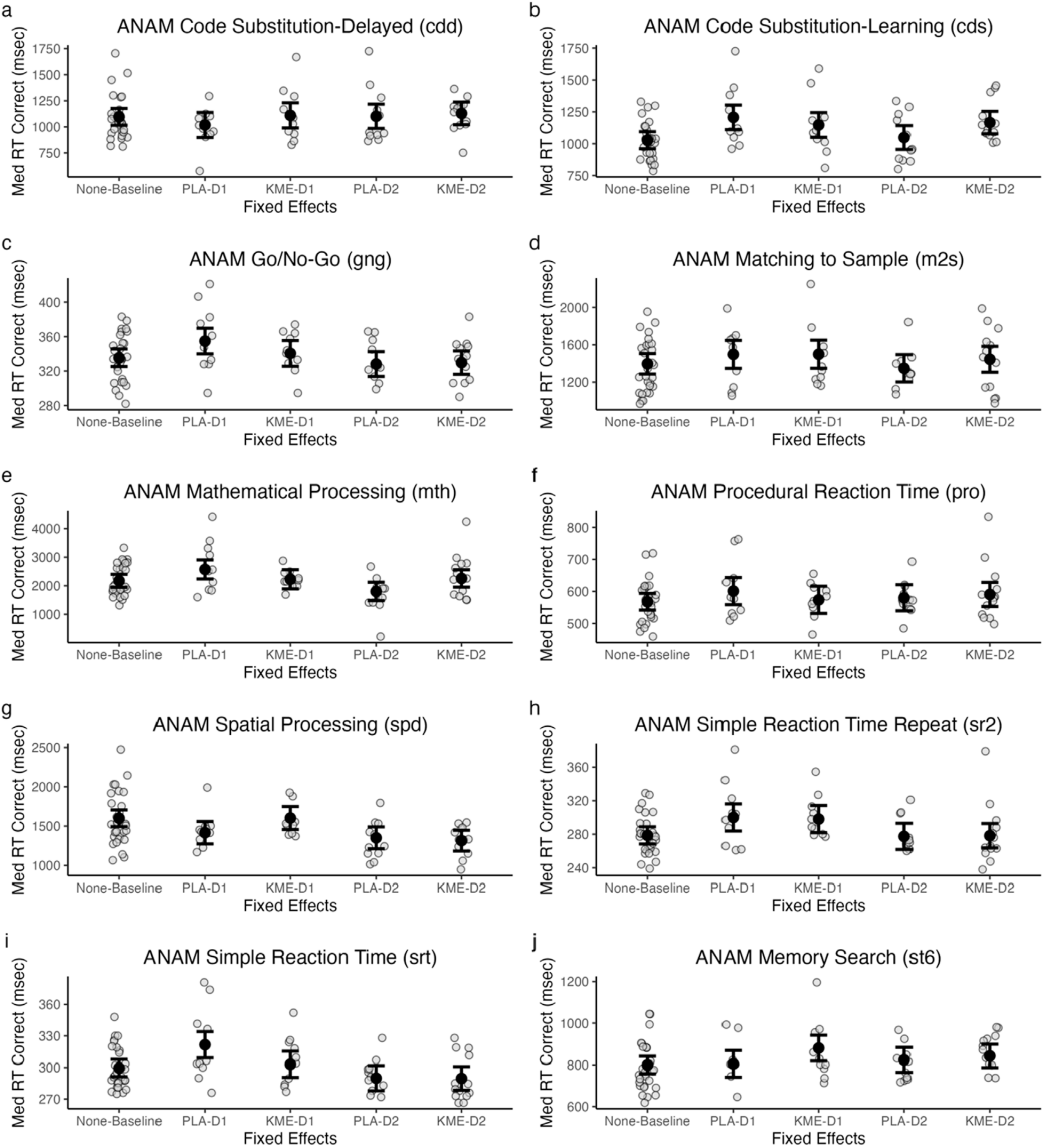
Model-estimated means of ANAM code substitution—delayed (CDD: **(A)**, code substitution—learning (CDS: **(B)**, go/no-go (GNG: **(C)**, matching to sample (M2S: **(D)**, mathematical processing (MTH: **(E)**, procedural reaction time (PRO: **(F)**, spatial processing (SPD: **(G)**, simple reaction time (SRT: **(H)**, simple reaction time—repeat (SR2: **(I)**, and memory search (ST6: **(J)** plotted by treatment and time point. All in milliseconds. PLA, placebo; KME, ketone monoester. Mountaineering day 1, D1; mountaineering day 2 = D2.

Figure 7 plots the median reaction time (ms) for correct trials in each test within ANAM-4, and Table 4 shows the test statistics.

**TABLE 4:** ANAM treatment × time point ANOVA model results.

Model	Predictor	*df*_*Num*_	*df*_*Den*_	*Sum sq*	*Mean sq*	*F*	*p*
CDS	Treatment	2	40.59	87,926.56	43,963.28	3.73	0.03*
	Time	1	40.36	48,176.93	48,176.93	4.09	0.049*
	Treatment × time	1	60.23	38,810.45	38,810.45	3.29	0.07^†^
CDD	Treatment	2	39.45	41,690.74	20,845.37	1.04	0.36
	Time	1	39.23	26,141.85	26,141.85	1.31	0.26
	Treatment × time	1	63.08	5,346.57	5,346.57	0.27	0.61
GNG	Treatment	2	40.83	946.82	473.41	1.61	0.21
	Time	1	40.61	3,481.36	3,481.36	11.86	0.001**
	Treatment × time	1	61.84	322.60	322.60	1.10	0.30
M2S	Treatment	2	40.23	23,524.60	11,762.30	0.46	0.63
	Time	1	40.00	100,960.01	100,960.01	3.98	0.052†
	Treatment × time	1	56.37	10,479.31	10,479.31	0.41	0.52
MTH	Treatment	2	45.25	270,263.45	135,131.73	1.01	0.37
	Time	1	45.00	1,361,644.38	1,361,644.38	10.14	0.003**
	Treatment × time	1	66.63	791,085.86	791,085.86	5.89	0.02*
PRO	Treatment	2	45.15	4,413.66	2,206.83	0.70	0.50
	Time	1	45.15	41.48	41.48	0.01	0.91
	Treatment × time	1	67.86	2,323.80	2,323.80	0.74	0.39
SPD	Treatment	2	36.50	860,848.45	430,424.22	20.22	<.001***
	Time	1	36.40	288,548.53	288,548.53	13.55	<.001***
	Treatment × time	1	50.58	56,108.87	56,108.87	2.64	0.11
SRT	Treatment	2	43.08	2,152.31	1,076.16	4.86	0.01*
	Time	1	42.89	5,073.01	5,073.01	22.89	<.001***
	Treatment × time	1	65.06	453.80	453.80	2.05	0.16
SR2	Treatment	2	44.13	10.44	5.22	0.01	0.99
	Time	1	44.03	4,520.87	4,520.87	10.68	0.002**
	Treatment × time	1	67.66	11.47	11.47	0.03	0.87
ST6	Treatment	2	36.32	36,802.19	18,401.09	3.65	0.04*
	Time	1	36.23	857.74	857.74	0.17	0.68
	Treatment × time	1	59.14	4,028.48	4,028.48	0.80	0.38

†p < .10, *p < .05, **p < .01, and ***p < .001.

#### 3.5.1 Code substitution—delayed

There were no significant main effects of treatment or time point and no significant interaction between these two variables.

#### 3.5.2 Code substitution—learning

There was a significant main effect of treatment, indicating that the median reaction time was faster in the PLA condition than in the KME condition. The main effect of time point was marginally significant. The interaction between these two variables was also marginally significant. *Post hoc t*-tests showed that while there were significant or marginally significant differences between baseline and placebo day 1 (t[46.54] = −3.94, *p* < 0.001), baseline and KME day 1 (t[47.10] = −2.62, *p* = 0.01), baseline and KME day 2 (t[46.47] = −3.39, *p* < 0.001), placebo day 1 and placebo day 2 (t[53.52] = 2.61, *p* = 0.01), KME day 1 and placebo day 2 (t[40.49] = 1.98, *p* = 0.05), and placebo day 2 and KME day 2 (t[54.26] = −2.05, *p* = 0.05), no significant difference was observed between baseline and placebo day 2 (t[47.09] = −0.48, *p* = 0.63), placebo day 1 and KME day 1 (t[53.28] = 0.96, *p* = 0.34), placebo day 1 and KME day 2 (t[40.21] = 0.86, *p* = 0.40), and KME day 1 and KME day 2 (t[53.97] = −0.32, *p* = 0.75).

#### 3.5.3 Go/no-go

The main effect of treatment was not significant. There was a significant main effect of time point, such that the median reaction time was faster on day 2 than on day 1. The interaction between these two variables was not significant.

#### 3.5.4 Matching to sample

The main effect of treatment was not significant. There was a marginally significant main effect of time point, such that the median reaction time was faster on day 2 than on day 1. The interaction between these two variables was not significant.

#### 3.5.5 Mathematical processing

The main effect of treatment was not significant. There was a significant main effect of time point, such that the median reaction time was faster on day 2 than on day 1. The interaction between these two variables was also significant. *Post hoc t*-tests showed that while there were significant or marginally significant differences between baseline and placebo day 1 (t[48.06] = −2.50, *p* = 0.02), baseline and placebo day 2 (t[48.61] = 2.41, *p* = 0.02), placebo day 1 and placebo day 2 (t[55.33] = 3.63, *p* < 0.001), placebo day 1 and KME day 2 (t[41.43] = 1.88, *p* = 0.07), KME day 1 and placebo day 2 (t[41.73] = 2.43, *p* = 0.02), and placebo day 2 and KME day 2 (t[56.08] = −2.27, *p* = 0.03), there was no significant difference between baseline and KME day 1 (t[48.63] = −0.34, *p* = 0.74), baseline and KME day 2 (t[47.94] = −0.57, *p* = 0.57), placebo day 1 and KME day 1 (t[55.09] = 1.59, *p* = 0.12), and KME day 1 and KME day 2 (t[55.78] = −0.14, *p* = 0.89).

#### 3.5.6 Procedural reaction time

The main effect of treatment was not significant. The main effect of time point was not significant. The interaction between these two variables was not significant.

#### 3.5.7 Spatial processing

The main effect of treatment was significant, such that PLA had a faster median reaction time than KME. There was also a significant main effect of time point, such that the median reaction time was faster on day 2 than on day 1. The interaction between these two variables was not significant.

#### 3.5.8 Simple reaction time—repeat

The main effect of treatment was not significant. There was a significant main effect of time point, such that the median reaction time was faster on day 2 than on day 1. The interaction between these two variables was not significant.

#### 3.5.9 Simple reaction time

The main effect of treatment was significant, such that KME had a faster median reaction time than PLA. There was also a significant main effect of time point, such that the median reaction time was faster on day 2 than on day 1. The interaction between these two variables was not significant.

#### 3.5.10 Memory search (ST6)

There was a significant main effect of treatment, such that PLA had a faster median reaction time than KME. The main effect of time point was not significant. The interaction between these two variables was not significant.

### 3.6 SWAY cognitive assessment

**FIGURE 8:**
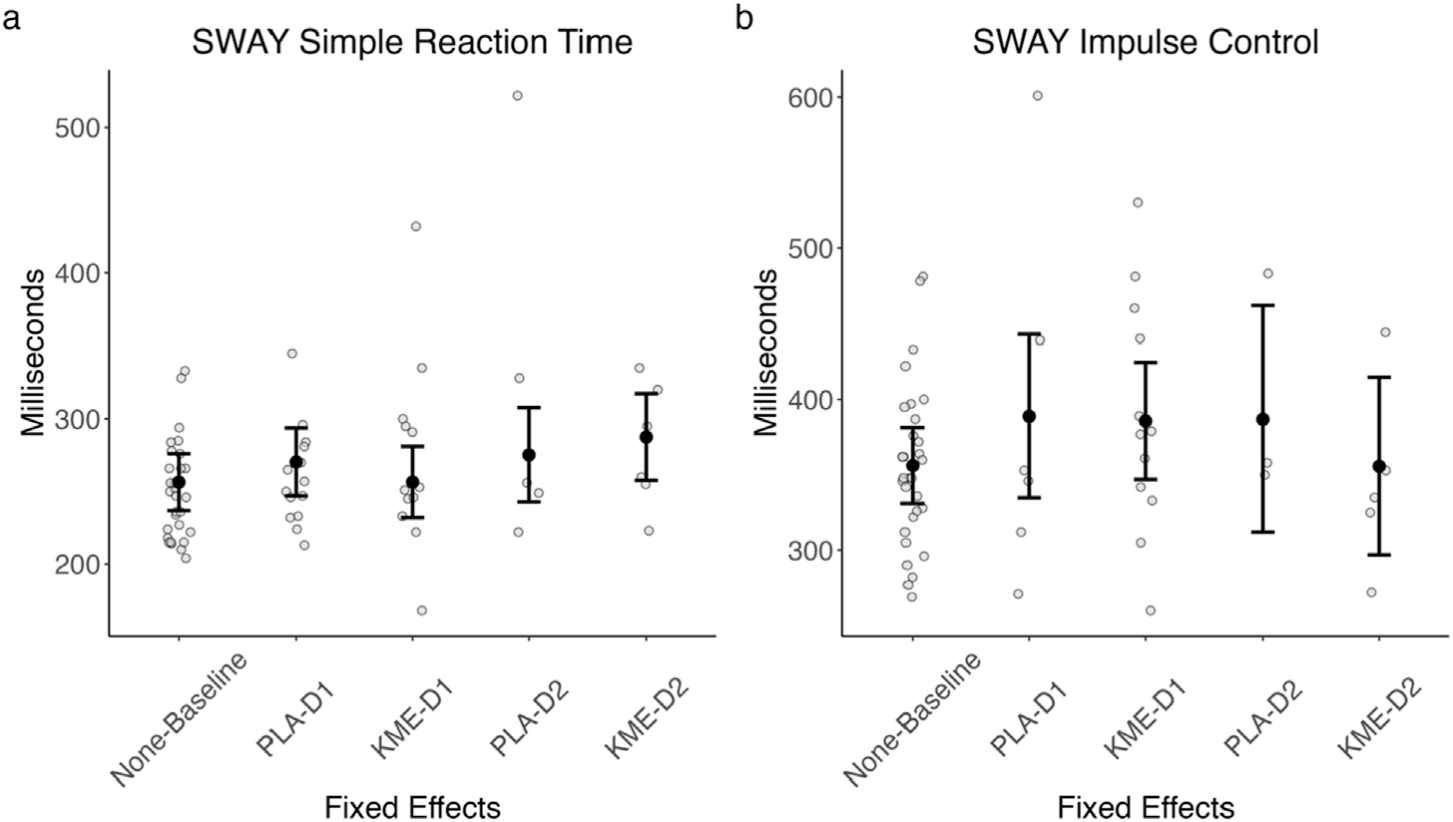
Model-estimated SWAY simple reaction time **(A)** and model-estimated impulse control **(B)** plotted by the treatment condition and time point. PLA, placebo; KME, ketone monoester. Mountaineering day 1, D1; mountaineering day 2, D2. The error bars denote 95% CI.

Figure 8 plots the median reaction time (ms) for correct trials in each test in the SWAY test, and Table 5 shows the test statistics. On the 2 mountaineering training days, SWAY was conducted near peak altitude shortly after completing the ascent portion of each training course.

**TABLE 5:** SWAY ANOVA model results.

Model	Predictor	*df*_*Num*_	*df*_*Den*_	*Sum sq*	*Mean sq*	*F*	*p*
Simple reaction time	Treatment	2	32.97	3,921.24	1,960.62	3.14	0.056^†^
	Time	1	32.50	2,081.98	2,081.98	3.33	0.08^†^
	Treatment × time	1	41.16	591.84	591.84	0.95	0.34
Impulse control	Treatment	2	35.07	2,100.00	1,050.00	0.32	0.73
	Time	1	34.04	1,176.26	1,176.26	0.36	0.55
	Treatment × time	1	50.15	689.70	689.70	0.21	0.65

^*†*^ *p < .*10*.*

#### 3.6.1 Simple reaction time

The main effect of treatment was marginally significant (F[3.140, 3.140] = 3.140, *p* = 0.056), such that PLA appeared to be associated with a faster median reaction time than KME particularly on day 2. There was also a marginally significant main effect of time point (F[3.335, 3.335] = 3.335, *p* = 0.077), such that the median reaction time was faster on day 1 than on day 2. The interaction between these two variables was not significant (F[0.948, 0.948] = 0.948, *p* = 0.336).

#### 3.6.2 Impulse control

The main effects of treatment and route were not significant. The interaction between these two variables was not significant.

### 3.7 Perceived effects of KME on gastrointestinal symptoms

Table 6 shows the test statistics of the chi-squared tests for each item on the GI symptom questionnaire. There were statistically significantly greater cases of heartburn, nausea, and vomiting, as well as marginally significantly greater cases of dizziness in KME than in PLA. There were no statistically significant differences in the incidents of bloating, intestinal cramps, abdominal pain, flatulence, diarrhea, headache, muscle cramps, or urge to urinate.

**TABLE 6:** Frequency of GI symptoms by the treatment conditions and the chi-square test results.

	Placebo (N = 28)	KME (N = 29)	Total (N = 57)	χ^2^	*p*
Heartburn				4.12	0.042*
No	26 (92.9%)	21 (72.4%)	47 (82.5%)		
Yes	2 (7.1%)	8 (27.6%)	10 (17.5%)		
Bloating				0.40	0.53
No	24 (85.7%)	23 (79.3%)	47 (82.5%)		
Yes	4 (14.3%)	6 (20.7%)	10 (17.5%)		
Nausea				7.17	0.007**
No	23 (82.1%)	14 (48.3%)	37 (64.9%)		
Yes	5 (17.9%)	15 (51.7%)	20 (35.1%)		
Vomiting				4.57	0.033*
No	25 (89.3%)	19 (65.5%)	44 (77.2%)		
Yes	3 (10.7%)	10 (34.5%)	13 (22.8%)		
Intestinal cramps				1.86	0.17
No	27 (96.4%)	25 (86.2%)	52 (91.2%)		
Yes	1 (3.6%)	4 (13.8%)	5 (8.8%)		
Abdominal pain				2.00	0.16
No	28 (100%)	27 (93.1%)	55 (96.5%)		
Yes	0 (0%)	2 (6.9%)	2 (3.5%)		
Flatulence				0.45	0.50
No	12 (42.9%)	15 (51.7%)	27 (47.4%)		
Yes	16 (57.1%)	14 (48.3%)	30 (52.6%)		
Diarrhea				0.67	0.41
No	26 (92.9%)	25 (86.2%)	51 (89.5%)		
Yes	2 (7.1%)	4 (13.8%)	6 (10.5%)		
Dizziness				3.04	0.081†
No	20 (71.4%)	26 (89.7%)	46 (80.7%)		
Yes	8 (28.6%)	3 (10.3%)	11 (19.3%)		
Headache				0.15	0.70
No	21 (75.0%)	23 (79.3%)	44 (77.2%)		
Yes	7 (25.0%)	6 (20.7%)	13 (22.8%)		
Muscle cramps				0.50	0.48
No	25 (89.3%)	24 (82.8%)	49 (86.0%)		
Yes	3 (10.7%)	5 (17.2%)	8 (14.0%)		
Urge to urinate				0.004	0.95
No	23 (82.1%)	24 (82.8%)	47 (82.5%)		
Yes	5 (17.9%)	5 (17.2%)	10 (17.5%)		

^†^ *p <* 0.10, *p < 0.05*,* and **p < 0.01.

### 3.8 Blood ketone and vomiting

Given the prevalence of the GI symptoms reported above, especially vomiting as it could eliminate the supplemented KME without being digested and metabolized, we conducted an analysis looking at the blood ketone level by the treatment condition (PLA vs. KME) and whether a given participant reported vomiting.

**FIGURE 9:**
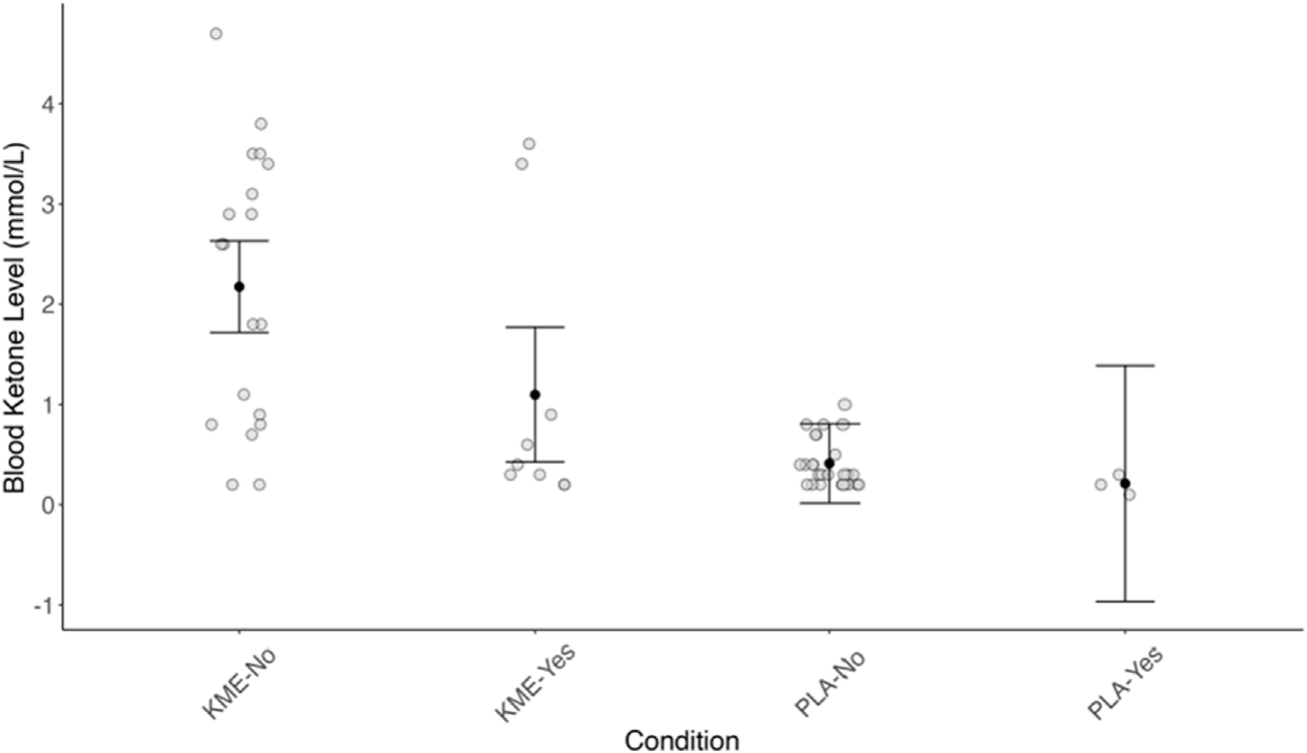
Blood ketone level (mmol/L) plotted by the incident of vomiting (no or yes) and the treatment condition (PLA or KME).

Figure 9 plots the blood ketone level by the incident of vomiting (no or yes) and the treatment condition (PLA vs. KME). There was a significant main effect of treatment (F[1, 40.621] = 13.85, *p* < 0.001). Among the individuals who reported no vomiting, those who consumed PLA had 1.33 mmol/L lower blood ketones than those who consumed KME (*p* < 0.001). There was no main effect of vomiting; however, the probability of no impact of vomiting on blood ketone levels was low (*p* = 0.08). Considering all individuals, those who consumed KME and did not report vomiting had a 1.08 mmol/L higher blood ketone level than those who did. There was no interaction between treatment and vomiting.

### 3.9 Vomiting as a potential moderator of the KME effect on cognitive performance

Given the prevalence of vomiting and its effect on the blood ketone level under the KME condition, as revealed in the previous section, we conducted analyses examining whether the vomiting status impacted the effects of KME on cognitive performance (as reported in Sections 3.5. and 3.6), our main target variables. These analyses (reported in Supplementary Table S3 for brevity) showed that the vomiting status had limited impact on the relationship between KME supplementation and cognitive performance on mountaineering training days. Specifically, in terms of the critical treatment–time point interaction, adding the vomiting status as a fixed-effect variable did not reveal the KME effect. The only two changes were (1) significant treatment–time point interaction in mathematical processing (which previously showed PLA being advantageous for preserving cognitive performance on mountaineering days) went away and (2) marginally significant treatment–time point interaction (*p* = .07) emerged in spatial processing, showing PLA being advantageous for preserving cognitive performance on mountaineering days. When interpreting these results, it should be noted that the blood ketone level was measured on top of the mountaineering training course only once, but the participants consumed KME (or PLA) five times (six doses, including the initial double dose; see Figure 1) throughout the mountaineering training days, including one right before the post-training data collection. Thus, the blood ketone level reported above is likely not perfectly reflective of participants’ blood ketone level throughout the training and/or during the cognitive tests.

## 4 Discussion

### 4.1 Summary of findings

We conducted an applied double-blind, placebo-controlled crossover trial examining the efficacy of KME on alpine training among military special operators. The participants were students and cadre in the US Army SOAMS run by the 10th Special Forces Group. To qualify for SOAMS, participants were required to first successfully complete the IFT covering 0.9 miles over 2,744 steps with 2,020 ft/615 m elevation gain (from 6,530 ft/2,012 m to 8,550 ft/2,606 m) and an average grade of 41% in under 45 min (average = 35.2 min). They went through 2 days of alpine training on two different courses, with several hours of training above 11,000 ft elevation; at peak ascent, the Spiral route reached 12,460 ft, and Blitzen route, 13,627 ft. The alpine training days were mentally and physically demanding and lasted for a long duration, i.e., 10 h and 14 h on average for the Spiral and Blitzen routes, respectively, and more than half of the time was spent at or above moderate- and vigorous-heart rate zones, as tracked by the Garmin Fenix 7X Pro Solar.

### 4.2 Cognitive performance in the mountaineering environment and sleep metrics

One unique aspect of the current study was the timing and environment at which we collected cognitive performance data. Specifically, we collected established measures of various kinds of cognitive performance on the day before the alpine training days at the basecamp (7,500 ft), as well as on 2 alpine training days near peak altitude and again at the basecamp level after training. The sleep metrics that were collected through the Oura Ring predicted this cognitive performance in the mountaineering environment well. Several sleep, heart rate, and heart rate variability metrics from the night before, such as respiration rate, sleep duration, lowest heart rate, and RMSSD during sleep, all significantly predicted cognitive performance in the mountaineering environment. Furthermore, the average of the sleep metrics over the 3 previous nights seems to provide additional predictive utility, as the 3-day averages of respiration rate, sleep duration, and the average and lowest heart rates during sleep were also found to be significantly correlated with cognitive performance in the mountaineering environment. These findings show the utility of sleep metrics in predicting cognitive performance in this challenging environment and emphasize the importance of considering trends over several nights vs. a single sleep cycle.

### 4.3 The effect of ketone monoester supplementation on high-altitude mountaineering training

Participants took six doses of KME or PLA 2–3 h apart throughout each alpine training day. Consistent with previous findings in laboratory studies (Evans et al., 2022; Prins et al., 2021), KME elevated the blood ketone level and decreased the blood glucose level. However, there was no clear indication that this increase in the blood ketone level brought physical or mental performance enhancement. Specifically, KME and PLA did not significantly differ in the distribution of time spent at each heart rate zone (light, moderate, and vigorous intensity, as calculated based on the American College of Sports Medicine’s training zones anchored to percentages of the age-predicted maximum heart rate). In other words, KME did not decrease the time spent in the more demanding heart rate zone. Furthermore, although not statistically significant, there was a trend toward participants on KME reporting greater perceived exertion, particularly on the Spiral route. Lack of a KME benefit on perceived exertion and fatigue could be due in part to participants having already adapted to the high-altitude environment as they had already completed a few training modules at high altitudes prior to the target training days of this experiment. As such, it is possible that the hypothesized benefits of the increased blood oxygenation of KME under hypoxic conditions might have been less pronounced among the current participants.

Regarding cognitive performance, a majority of outcome measures (9 out of 12) showed no significant difference between KME and PLA. While one variable showed a favorable effect of KME (ANAM simple reaction time at the post-training data collection), two outcome measures favored PLA (ANAM spatial processing and mathematical processing at the post-training data collection). In our laboratory-based experiments, KME attenuated the decreases in SpO_2_ and cognitive performance under severe hypoxia at rest (McClure, et al., 2024a), but when cognitive performance was measured under hypoxia in conjunction with moderate-intensity exercise, the attenuated decrease in SpO_2_ with KME was not accompanied by a cognitive performance benefit (McClure et al., 2024b, Philips, Kernagis, et al., in press). The lack of a KME advantage on cognitive performance in hypoxia with moderate-intensity exercise is likely due to the potency of acute exercise on cognitive performance, essentially trumping other potential mediators. It should also be pointed out that, to support the hypoxic exercise bout, in our laboratory experiment, both the KME and PLA treatments were supplemented with glucose (in the form of dextrose and maltodextrin). The SOAMS special operators in the current field study were clearly exercising at a moderate-to-high intensity for several hours. Food intake was not controlled nor inhibited, and the treatment conditions were not calorie-matched; thus, consumption of carbohydrate, fat, and protein calories throughout the mountaineering training days certainly could have influenced the outcomes. Furthermore, the potential influence of vomiting and other gastrointestinal symptoms due to KME cannot be ruled out (see below).

### 4.4 Caution on gastrointestinal symptoms and vomiting

There was a higher incidence of GI symptoms (heartburn, nausea, and vomiting) when taking KME compared to PLA. Ingestion of exogenous ketone ester often produces GI symptoms (Clarke et al., 2012; Evans and Egan, 2018). GI symptoms likely create distraction and hindrance to performance, and vomiting soon after ingestion can certainly compromise the intended dosing (see our results on vomiting and blood ketone levels). As such, future research should try to identify the dosing amount and timing that can elevate blood ketone levels with minimal side effects.

### 4.5 Limitations and overall conclusion

This challenging field experiment was generally successful as we were able to collect useful data (cognitive performance data, blood ketone and glucose levels, perceived effort, and fatigue) at key time points, including near the highest altitudes of each ascent, with special operators performing intensive and long-duration mountaineering training—much of it above 11,000 ft and in the moderate-to-high-intensity training zone (based on continuous heart rate collection). As with any field-based experiment in a real-world training environment, there are limitations that affect rigor and reproducibility: (i) by the nature of the training course, the KME vs. PLA crossover design was complicated by two different mountaineering routes, and, although we attempted to reduce the impact via randomization up-front and mixed models in the analysis, a standardized route repeated on both test days would have likely reduced extraneous sources of variability; (ii) we did not control dietary intake before, during, or after mountaineering training, which could have influenced the outcomes by introducing another source of inter-individual response heterogeneity; (iii) we were unsuccessful in collecting SpO_2_ at the highest altitudes due to inconsistent pulse oximeter readings as sensor reliability encountered problems above 8,000 ft; and (iv) some participants experienced GI side effects, including vomiting, which led to high variability in blood ketone levels.

These limitations notwithstanding, from the available data, we conclude that there were no clear benefits of KME supplementation on physiologic demand, perceived effort, fatigue, or cognitive performance during intensive and long-duration mountaineering training at high altitudes among SOF personnel.

## Data Availability

The raw data supporting the conclusion of this article will be made available by the authors, without undue reservation.

## Supplementary material

SUPPLEMENTARY FIGURE S1 Change in elevation plotted by time during the incline fitness test from the Garmin watch.

SUPPLEMENTARY FIGURE S2 Heart rate data tracings during the inclined fitness test for N = 24 participants. Heart rate zones are defined by the American College of Sports Medicine defined zones and are calculated based on the percentage of age-predicted maximum heart rate. Very light (no color): <57% HR_max_; light (green): 57%–63%; moderate (yellow): 64%–76%; vigorous (orange): 77%–95%; maximal (red): >95%.

SUPPLEMENTARY FIGURE S3 Raw heart rate during the Blitzen route. Heart rate zones are defined by the American College of Sports Medicine defined zones and are calculated based on the percentage of age-predicted heart rate max. Very light (no color): <57% HR_max_; light (green): 57%–63%; moderate (yellow): 64%–76%; vigorous (orange): 77%–95%; maximal (red): >95%.

SUPPLEMENTARY FIGURE S4 Raw heart rate during the Spiral route. Heart rate zones are defined by the American College of Sports Medicine defined zones and are calculated based on the percentage of age-predicted heart rate max. Very light (no color): <57% HR_max_; light (green): 57%–63%; moderate (yellow): 64%–76%; vigorous (orange): 77%–95%; maximal (red): >95%.

SUPPLEMENTARY FIGURE S5 Model-estimated means of ANAM code substitution—delayed [CDD: **(A)**], code substitution—learning [CDS: **(B)**], go/no-go [GNG: **(C)**], matching to sample [M2S: **(D)**], mathematical processing [MTH: **(E)**], procedural reaction time [PRO: **(F)**], spatial processing [SPD: **(G)**], simple reaction time [SRT: **(H)**], simple reaction time—repeat [SR2: **(I)**], and memory search [ST6: **(J)**] plotted by treatment and route, all in milliseconds. PLA, placebo; KME, ketone monoester. Mountaineering day 1 = D1; mountaineering day 2 = D2.

SUPPLEMENTARY FIGURE S6 Model-estimated SWAY simple reaction time **(A)** and model-estimated impulse control **(B)** plotted by the treatment condition and route. PLA, placebo; KME, ketone monoester. Mountaineering day 1 = D1; mountaineering day 2 = D2. The error bars denote 95% CI.

SUPPLEMENTARY FIGURE S7 Model-estimated means of Borg RPE **(A)** and Samn–Perelli Fatigue Scale **(B)** ratings plotted by the treatment condition and route. PLA, placebo; KME, ketone monoester. The error bars denote 95% CI.

SUPPLEMENTARY TABLE S1 Results of one-way analysis of variance (ANOVA) examining the effect of time point (baseline, alpine training day 1, and day 2) on each sleep and readiness metric.

SUPPLEMENTARY TABLE S2 Cognitive test treatment × route ANOVA model results.

SUPPLEMENTARY TABLE S3 Subjective questionnaire treatment × time point ANOVA model results.

SUPPLEMENTARY TABLE S4 Cognitive test treatment × time with vomiting ANOVA model results.
