# Supplementary material for: Effect of ketone monoester supplementation on elite operators’ mountaineering training
Source: Front Physiol. 2024 Sep 3;15:1411421. doi: 10.3389/fphys.2024.1411421 (PMC11405315; doi:10.3389/fphys.2024.1411421)
Supplement: Supplementary file 1 [file Image1.pdf]

Supplemental Materials

Supplemental Table 1. Results of one-way analysis of variance (ANOVA) examining the effect of Time Point (Baseline, Alpine Training Day 1, and Day 2) on each sleep and readiness metric.

Dependent Variable	Predictor	Sum of Squares	*df*	Mean Square	*F*	*p*
**readiness_score**	Time Point	4416.991	2	2208.495	29.80	< .001***
	Residuals	4298.944	58	74.12		
**sleep_score**	Time Point	3079.715	2	1539.857	11.00	< .001***
	Residuals	8259.705	59	139.995		
**hr_lowest**	Time Point	199.298	2	99.649	1.20	0.308
	Residuals	4895.541	59	82.975		
**hr_average**	Time Point	492.403	2	246.202	4.62	0.014*
	Residuals	3145.89	59	53.32		
**r_mssd**	Time Point	4829.295	2	2414.647	3.38	0.041*
	Residuals	41493.689	58	715.408		
**breath_average**	Time Point	9.837	2	4.919	2.04	0.14
	Residuals	142.603	59	2.417		
**hr_lowest_3day**	Time Point	1.401	2	0.701	0.02	0.982
	Residuals	2304.986	60	38.416		
**hr_average_3day**	Time Point	17.002	2	8.501	0.17	0.842
	Residuals	2949.032	60	49.151		
**r_mssd_3day**	Time Point	756.339	2	378.169	0.65	0.524
	Residuals	34768.501	60	579.475		
**breath_average_3da**	Time Point	1.743	2	0.871	0.48	0.621
	Residuals	109.033	60	1.817		
**duration_min**	Time Point	296623.646	2	148311.823	21.53	< .001***
	Residuals	406404.241	59	6888.207		
**duration_min_3day**	Time Point	99618.123	2	49809.062	15.06	< .001***
	Residuals	198441.202	60	3307.353		

$*p < .05$, $***p < .001$

Supplemental Figure 1. Change in elevation plotted by time during the incline fitness test from the Garmin watch.

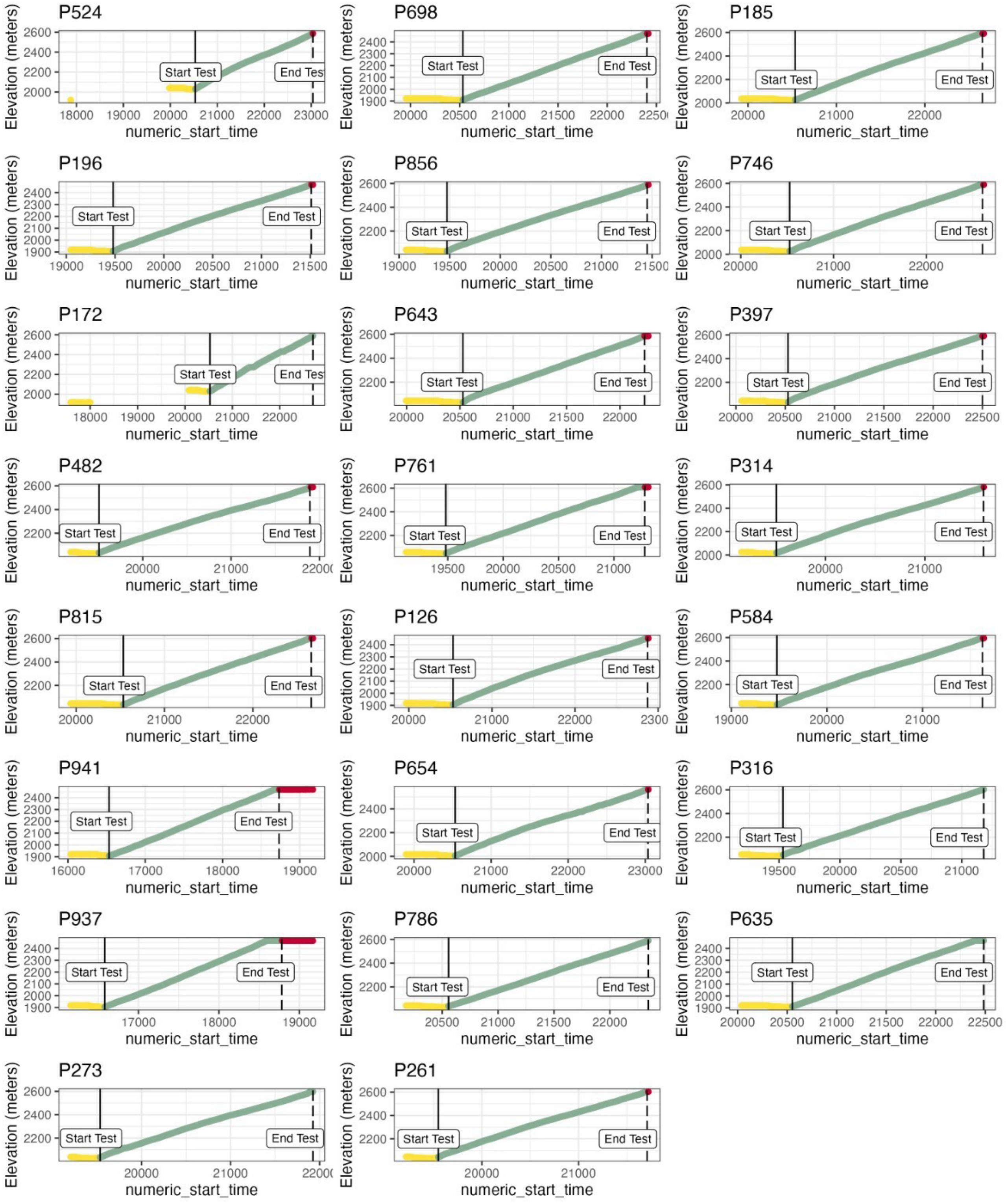

Supplemental Figure 2. Heart rate data tracings during the inclined fitness test for N=24 participants. Heart rate zones are defined by American College of Sports Medicine defined zones and are calculated based on percentage of age-predicted maximum heart rate. Very Light (No Color): < 57% HR_{max}, Light (Green): 57-63 %, Moderate (Yellow): 64-76%, Vigorous (Orange): 77-95%, Maximal (Red): >95%.

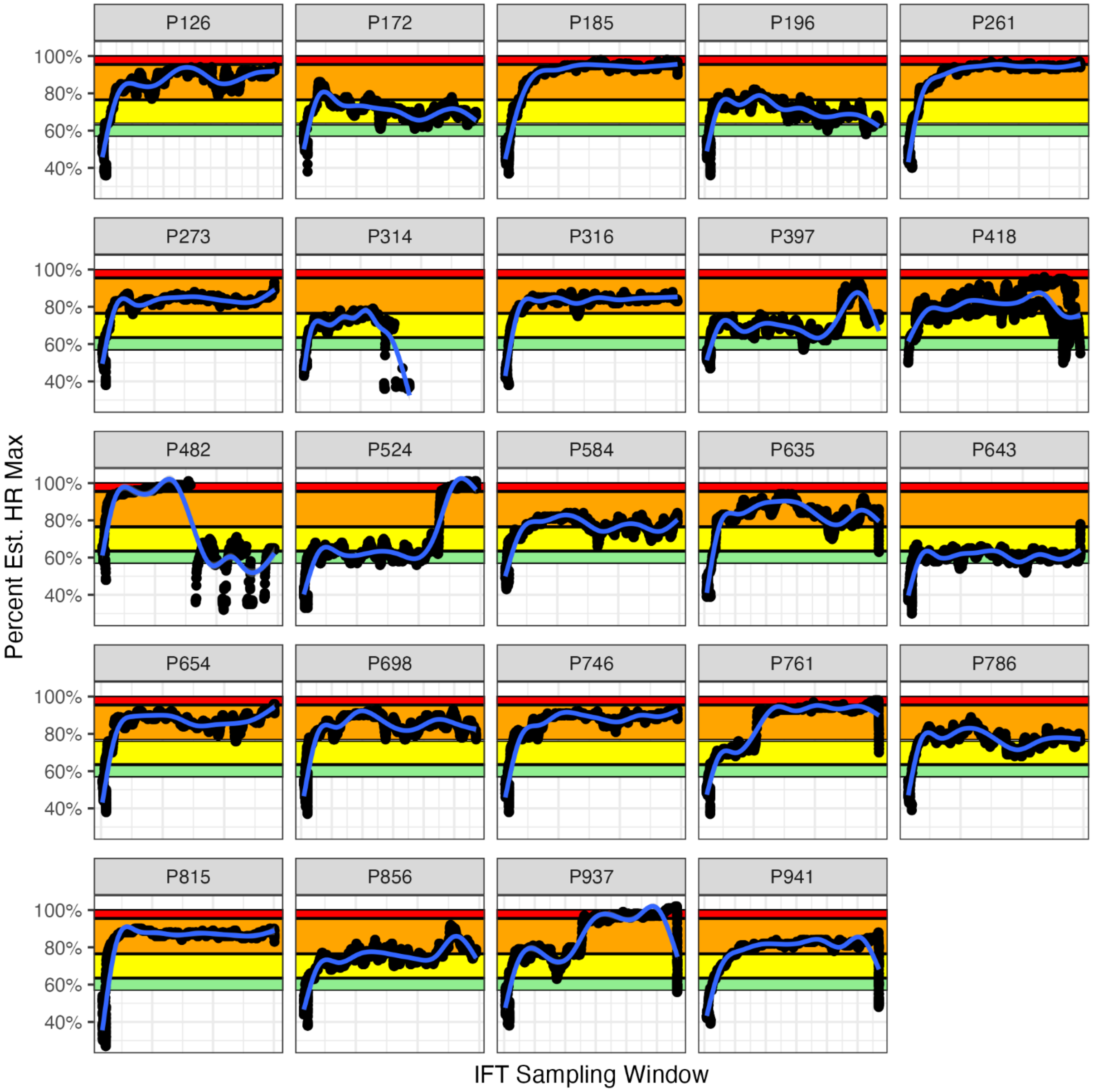

Supplemental Figure 3. Raw heart rate during the Blitzen route. Heart rate zones are defined by American College of Sports Medicine defined zones and are calculated based on percentage of age-predicted heart rate max. Very Light (No Color): < 57% HR_{max}, Light (Green): 57-63 %, Moderate (Yellow): 64-76%, Vigorous (Orange): 77-95%, Maximal (Red): >95%.

Blitzen (peak elevation 13,627 ft).

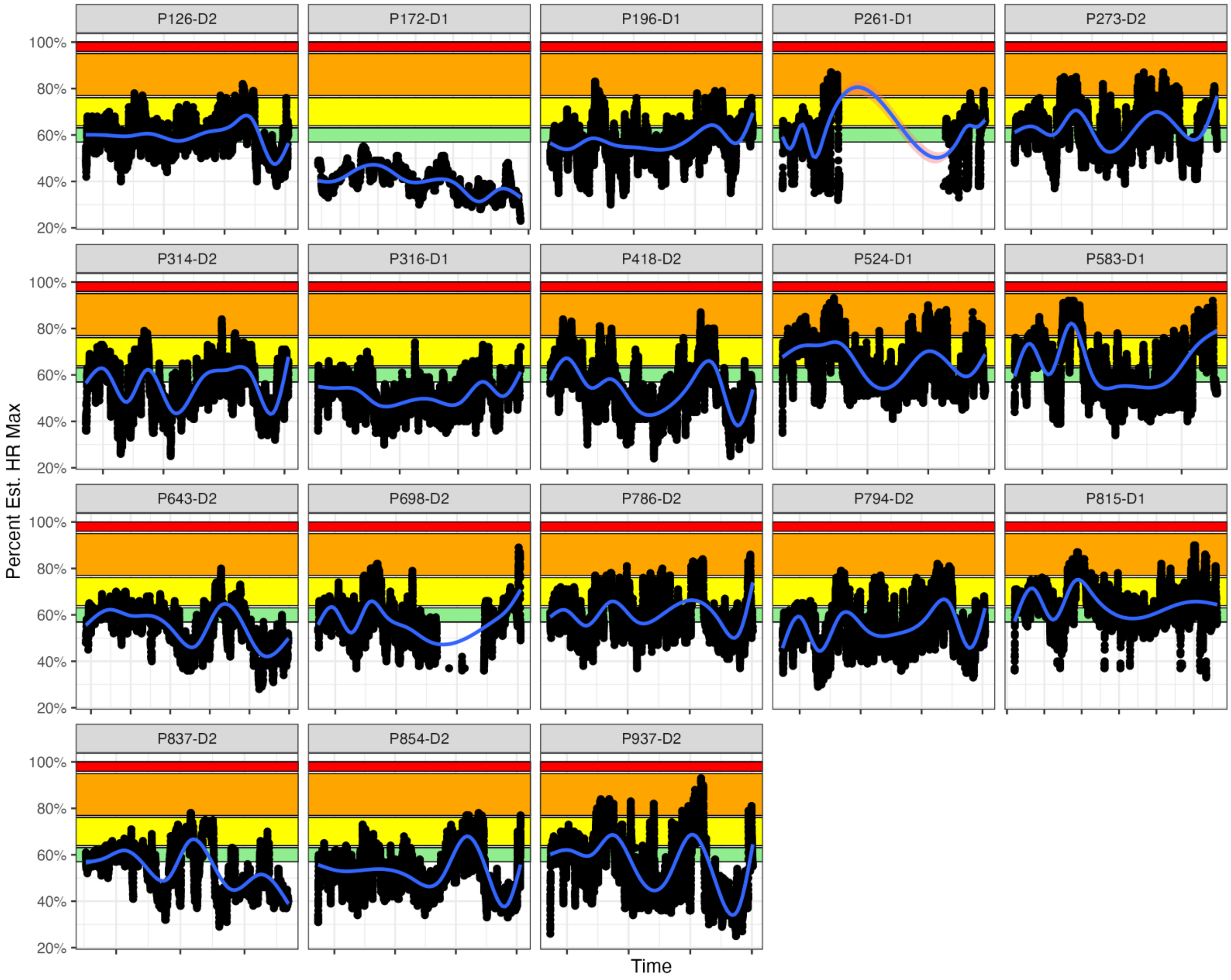

Supplemental Figure 4. Raw heart rate during the Spiral route. Heart rate zones are defined by American College of Sports Medicine defined zones and are calculated based on percentage of age-predicted heart rate max. Very Light (No Color): < 57% HR_{max}, Light (Green): 57-63 %, Moderate (Yellow): 64-76%, Vigorous (Orange): 77-95%, Maximal (Red): >95%.

Spiral (peak elevation 12,460 ft)

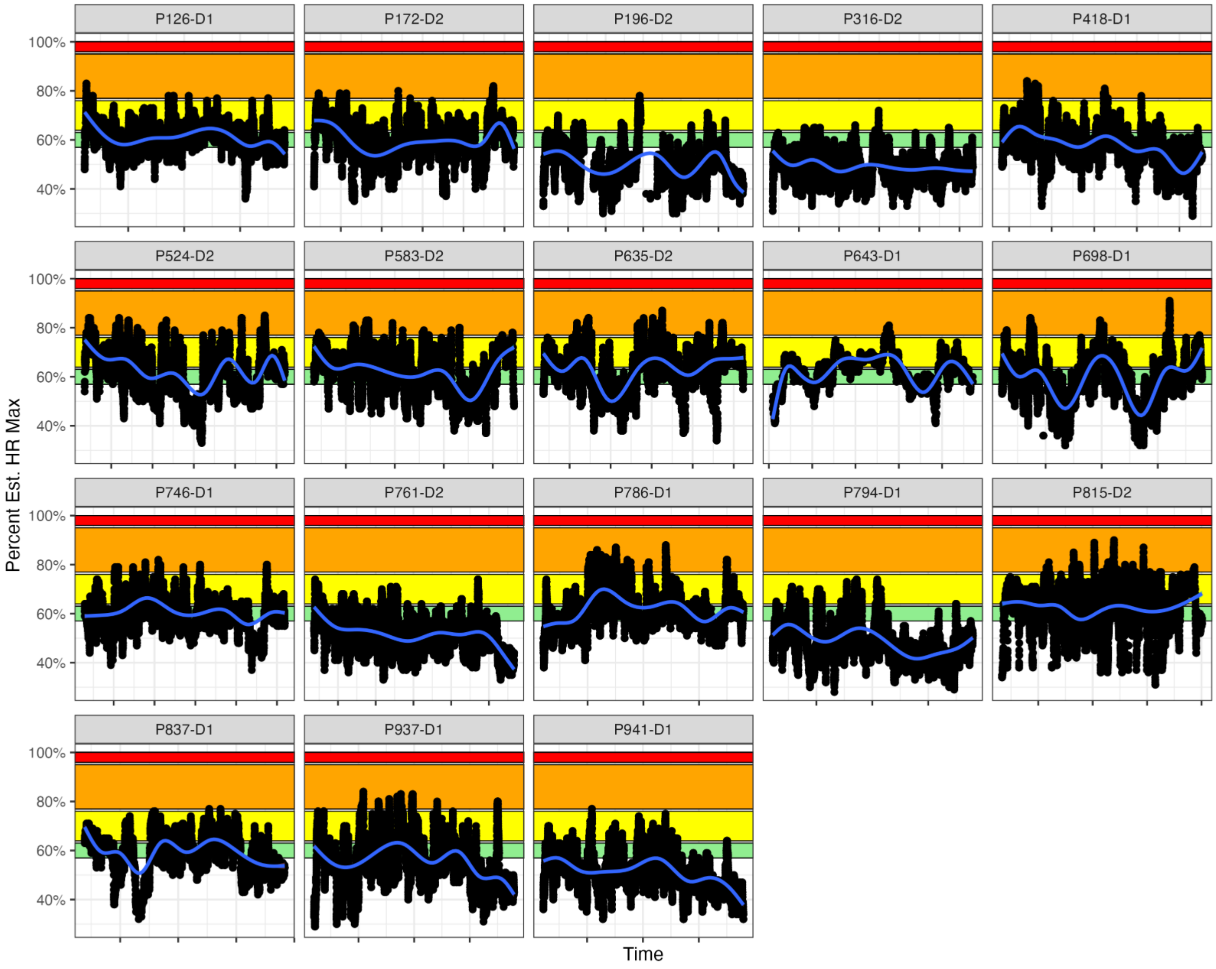

Supplemental Figure 5. Model-estimated means of ANAM Code Substitution-Delayed (CDD: panel a),Code Substitution-Learning (CDS: panel b), Go/No-Go (GNG: panel c), Matching to Sample (M2S: panel d), Mathematical Processing (MTH: panel e), Procedural Reaction Time (PRO: panel f), Spatial Processing (SPD: panel g), Simple Reaction Time (SRT: panel h), Simple Reaction Time-Repeat (SR2: panel i), and Memory Search (ST6: panel j) plotted by treatment and route. All in milliseconds. PLA = placebo, KME = ketone monoester. Mountaineering day 1 = D1, mountaineering day 2 = D2.

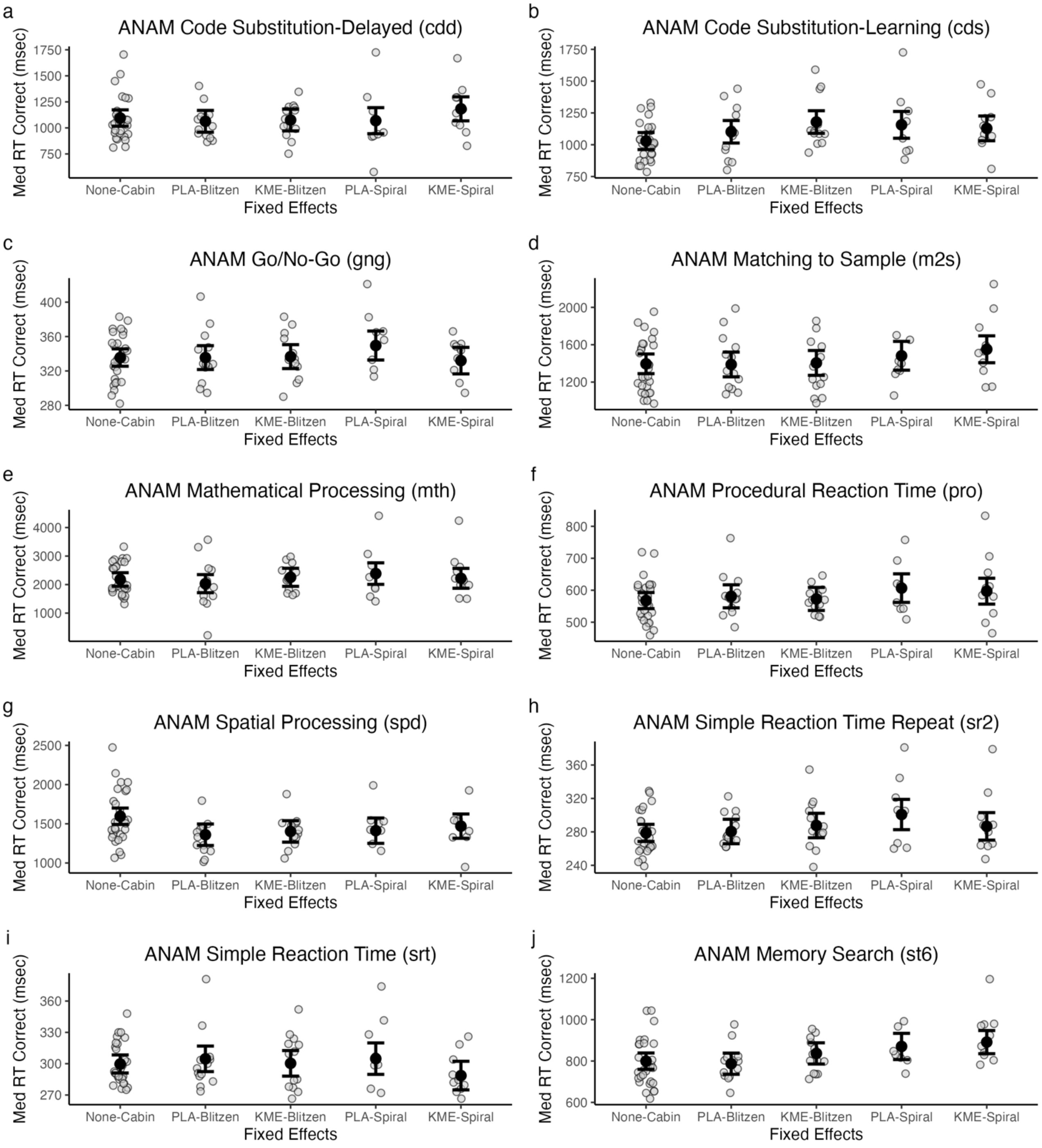

Supplemental Figure 6. Model-estimated SWAY simple reaction time (panel a) and model-estimated impulse control (panel b) plotted by the treatment condition and route. PLA = placebo, KME = ketone monoester. Mountaineering day 1 = D1, mountaineering day 2 = D2. The error bars denote 95% CI.

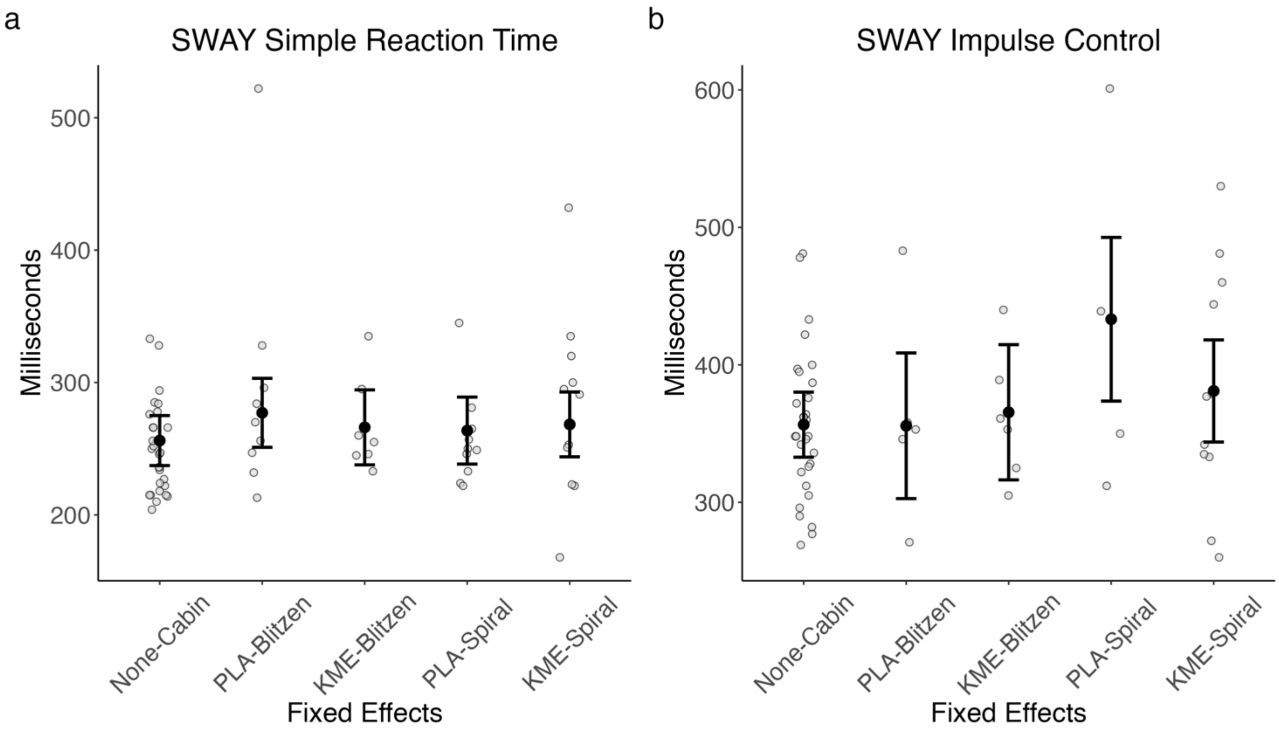

Supplemental Table 2. Cognitive Tests Treatment × Route ANOVA Model Results

Model	Predictor	df_{Num}	df_{Den}	*Sum sq*	*Mean sq*	*F*	*p*
CDS	Treatment	2	40.00	51533.06	25766.53	1.32	0.28
	Route	1	39.06	31188.01	31188.01	1.60	0.21
	Treat.×Route	1	62.40	12931.88	12931.88	0.66	0.42
CDD	Treatment	2	41.53	135151.73	67575.87	5.05	0.01*
	Route	1	40.66	59.55	59.55	0.00	0.95
	Treat.×Route	1	61.75	13515.57	13515.57	1.01	0.32
GNG	Treatment	2	42.30	884.75	442.37	1.16	0.32
	Route	1	41.25	222.23	222.23	0.58	0.45
	Treat.×Route	1	65.93	476.12	476.12	1.24	0.27
M2S	Treatment	2	40.02	164313.51	82156.75	3.30	0.05†
	Route	1	39.33	136952.15	136952.15	5.50	0.02*
	Treat.×Route	1	55.96	3737.21	3737.21	0.15	0.70
MTH	Treatment	2	43.07	165672.37	82836.19	0.47	0.63
	Route	1	42.19	247055.73	247055.73	1.41	0.24
	Treat.×Route	1	62.75	188521.68	188521.68	1.08	0.30
PRO	Treatment	2	46.07	12203.53	6101.76	1.98	0.15
	Route	1	44.87	6235.65	6235.65	2.03	0.16
	Treat.×Route	1	67.95	1.53	1.53	0.00	0.98
SPD	Treatment	2	36.55	242391.24	121195.62	3.93	0.03*
	Route	1	36.07	33476.15	33476.15	1.09	0.30
	Treat.×Route	1	57.05	217.15	217.15	0.01	0.93
SRT	Treatment	2	45.91	2316.55	1158.27	2.30	0.11
	Route	1	44.71	935.99	935.99	1.86	0.18
	Treat.×Route	1	67.98	705.38	705.38	1.40	0.24
SR2	Treatment	2	45.14	1178.35	589.17	1.75	0.19
	Route	1	43.98	319.74	319.74	0.95	0.34
	Treat.×Route	1	67.93	222.01	222.01	0.66	0.42
ST6	Treatment	2	36.52	79975.20	39987.60	9.51	<.001***
	Route	1	35.76	43769.87	43769.87	10.41	0.003**
	Treat.×Route	1	56.19	853.18	853.18	0.20	0.65
Simple Reaction Time	Treatment	2	34.21	1157.37	578.69	0.80	0.46
	Route	1	34.46	241.36	241.36	0.33	0.57
	Treat.×Route	1	44.37	280.82	280.82	0.39	0.54
Impulse Control	Treatment	2	34.38	18660.89	9330.45	3.28	0.05†
	Route	1	34.62	10487.25	10487.25	3.69	0.06†
	Treat.×Route	1	48.40	3443.98	3443.98	1.21	0.28

† $p < .10$, * $p < .05$, ** $p < .01$, *** $p < .001$

Supplemental Figure 7. Model-estimated means of Borg RPE (panel a) and Samn-Perelli Fatigue Scale (panel b) ratings plotted by the treatment condition and the route, where PLA = placebo & KME = ketone monoester. The error bars denote 95% CI.

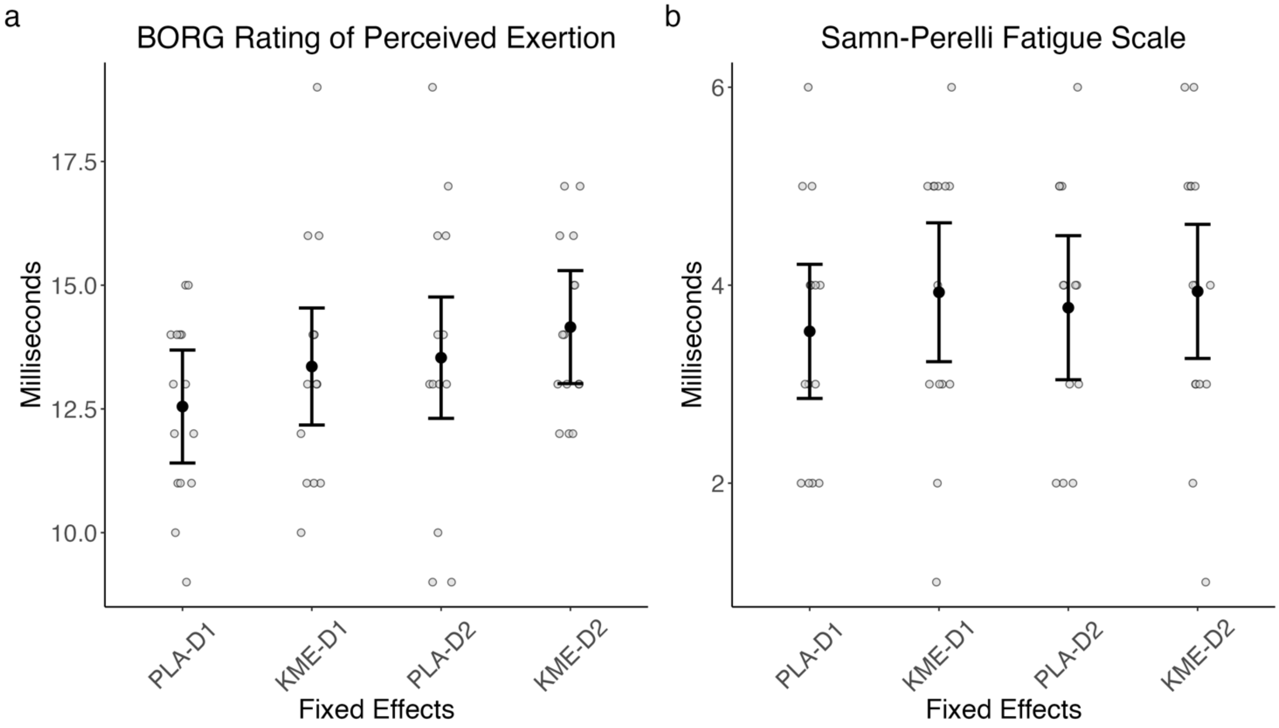

Supplemental Table 3. Subjective Questionnaires Treatment × Time Point ANOVA Model Results

Model	Predictor	df_{Num}	df_{Den}	*Sum sq*	*Mean sq*	*F*	*p*
Perceived Exertion	Treatment	1	27.47	7.18	7.18	1.56	0.22
	Time	1	27.47	11.21	11.21	2.44	0.13
	Treat.×Time	1	27.84	0.10	0.10	0.02	0.88
Perceived Fatigue	Treatment	1	27.61	1.11	1.11	0.63	0.44
	Time	1	27.61	0.22	0.22	0.12	0.73
	Treat.×Time	1	27.74	0.17	0.17	0.10	0.76

Supplemental Table 3. Cognitive Tests Treatment × Time with Vomiting ANOVA Model Results

Model	Predictor	df_{Num}	df_{Den}	*Sum sq*	*Mean sq*	*F*	*p*
CDS	Treatment	2	44.17	6426.65	6426.65	0.60	0.44
	Time	1	44.41	172294.18	86147.09	7.99	0.001**
	Vomiting	1	48.83	210.40	210.40	0.02	0.89
	Treat.×Time	1	63.44	36548.35	36548.35	3.39	0.07†
CDD	Treatment	2	42.95	50202.47	50202.47	2.85	0.10†
	Time	1	43.19	45582.33	22791.17	1.29	0.28
	Vomiting	1	48.59	18931.63	18931.63	1.07	0.31
	Treat.×Time	1	65.50	11617.83	11617.83	0.66	0.42
GNG	Treatment	2	44.35	113.16	113.16	0.45	0.51
	Time	1	44.59	3713.36	1856.68	7.31	0.002**
	Vomiting	1	49.16	534.19	534.19	2.10	0.15
	Treat.×Time	1	64.04	101.53	101.53	0.40	0.53
M2S	Treatment	2	43.74	233.08	233.08	0.01	0.91
	Time	1	43.95	81501.27	40750.63	2.06	0.14
	Vomiting	1	46.77	139645.48	139645.48	7.05	0.01*
	Treat.×Time	1	57.40	42337.56	42337.56	2.14	0.15
MTH	Treatment	2	44.59	203110.09	203110.09	1.82	0.18
	Time	1	44.82	1722791.32	861395.66	7.70	0.001**
	Vomiting	1	48.57	773550.61	773550.61	6.92	0.01*
	Treat.×Time	1	61.56	280148.88	280148.88	2.51	0.12
PRO	Treatment	2	48.88	252.08	252.08	0.09	0.77
	Time	1	48.99	3282.40	1641.20	0.58	0.57
	Vomiting	1	58.53	888.70	888.70	0.31	0.58
	Treat.×Time	1	72.99	1251.14	1251.14	0.44	0.51
SPD	Treatment	2	39.94	62806.05	62806.05	3.28	0.08†
	Time	1	40.28	481242.84	240621.42	12.58	<.001***
	Vomiting	1	43.40	9380.65	9380.65	0.49	0.49
	Treat.×Time	1	54.14	65088.39	65088.39	3.40	0.07†
SRT	Treatment	2	46.73	630.14	630.14	3.15	0.08†
	Time	1	46.96	5328.54	2664.27	13.32	<.001***
	Vomiting	1	52.69	73.98	73.98	0.37	0.55
	Treat.×Time	1	68.43	316.09	316.09	1.58	0.21
SR2	Treatment	2	47.55	8.89	8.89	0.02	0.88
	Time	1	47.73	5348.84	2674.42	7.05	0.002**
	Vomiting	1	55.36	178.10	178.10	0.47	0.50
	Treat.×Time	1	71.72	0.56	0.56	0.00	0.97
ST6	Treatment	2	39.72	11317.92	11317.92	2.63	0.11
	Time	1	40.40	18377.64	9188.82	2.14	0.13
	Vomiting	1	45.76	9592.74	9592.74	2.23	0.14
	Treat.×Time	1	60.55	1344.24	1344.24	0.31	0.58

Simple Reaction Time	Treatment	2	36.78	2.30	2.30	0.00	0.95
	Time	1	36.74	3190.31	1595.16	2.86	0.07†
	Vomiting	1	40.28	0.17	0.17	0.00	0.99
	Treat.×Time	1	44.76	654.53	654.53	1.17	0.28
Impulse Control	Treatment	2	36.41	3914.87	3914.87	1.60	0.21
	Time	1	37.98	740.36	370.18	0.15	0.86
	Vomiting	1	50.15	20718.08	20718.08	8.46	0.01**
	Treat.×Time	1	53.87	15.33	15.33	0.01	0.94

† $p < .10$, * $p < .05$, ** $p < .01$, *** $p < .001$
